# Synthesis, Structure and Antimicrobial Properties of Novel Benzalkonium Chloride Analogues with Pyridine Rings

**DOI:** 10.3390/molecules22010130

**Published:** 2017-01-13

**Authors:** Bogumił Brycki, Izabela Małecka, Anna Koziróg, Anna Otlewska

**Affiliations:** 1 Laboratory of Microbiocides Chemistry, Faculty of Chemistry, Adam Mickiewicz University in Poznan, Umultowska 89b, 61-614 Poznań, Poland; imalecka@amu.edu.pl; 2 Institute of Fermentation Technology and Microbiology, Faculty of Biotechnology and Food Sciences, Lodz University of Technology, Wólczańska 171/173, 90-924 Łódź, Poland; anna.kozirog@p.lodz.pl (A.K.); anna.otlewska@p.lodz.pl (A.O.)

**Keywords:** quaternary ammonium salts, benzalkonium chlorides, biocides, antimicrobial resistance, minimal inhibitory concentration, critical micellization concentrations

## Abstract

Quaternary ammonium compounds (QACs) are a group of compounds of great economic significance. They are widely used as emulsifiers, detergents, solubilizers and corrosion inhibitors in household and industrial products. Due to their excellent antimicrobial activity QACs have also gained a special meaning as antimicrobials in hospitals, agriculture and the food industry. The main representatives of the microbiocidal QACs are the benzalkonium chlorides (BACs), which exhibit biocidal activity against most bacteria, fungi, algae and some viruses. However, the misuses of QACs, mainly at sublethal concentrations, can lead to an increasing resistance of microorganisms. One of the ways to avoid this serious problem is the introduction and use of new biocides with modified structures instead of the biocides applied so far. Therefore new BAC analogues **P13**–**P18** with pyridine rings were synthesized. The new compounds were characterized by NMR, FT-IR and ESI-MS methods. PM3 semiempirical calculations of molecular structures and the heats of formation of compounds **P13**–**P18** were also performed. Critical micellization concentrations (CMCs) were determined to characterize the aggregation behavior of the new BAC analogues. The antimicrobial properties of novel QACs were examined by determining their minimal inhibitory concentration (MIC) values against the fungi *Aspergillus niger*, *Candida albicans*, *Penicillium chrysogenum* and bacteria *Staphylococcus aureus*, *Bacillus subtilis*, *Escherichia coli* and *Pseudomonas aeruginosa*. The MIC values of *N*,*N*-dimethyl-*N*-(4-methylpyridyl)-*N*-alkylammonium chlorides for fungi range from 0.1 to 12 mM and for bacteria, they range from 0.02 to 6 mM.

## 1. Introduction

Quaternary ammonium compounds (QACs) are a group of cationic surfactants widely used in household, agricultural, clinical and industrial products. QACs are surface active organic compounds containing at least one long hydrocarbon substituent attached covalently to a positively charged quaternary nitrogen atom. The other substituents can be alkyl, aromatic, ether or ester groups. Owing to the varied structures and a wide span of hydrophilic–lipophilic balance QACs are extensively used as emulsifiers [1], dispersants [2,3], fabric softeners [4], cosmetic ingredients [5] and phase transfer catalysts [6,7], gene delivery agents [8], antimicrobial [9,10] and anticorrosion [11,12] agents.

**Figure 1:**
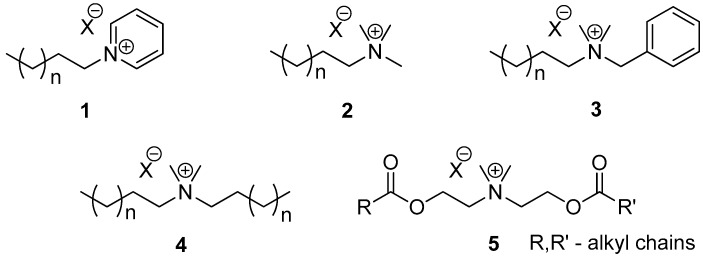
Examples of antimicrobial quaternary ammonium halides: alkylpyridinium (**1**); alkyltrimethylammonium (**2**); benzalkonium (**3**); dialkyldimethylammonium (**4**) and diesteralkonium (**5**).

Among a large number of cationic surfactants, examples of which are shown in Figure 1, benzalkonium chlorides **3** deserve the special attention due to their unique biocidal properties. They were presented for the first time as antimicrobial agents by Domagk in 1935 [13]. Nowadays, they are widely used as disinfectants and antiseptics, as well as preservatives for ophthalmic, nasal and inhalational drugs. They are also active substances in medicines for throat and mouth infections. The mechanism of their biocidal action toward most bacteria, fungi and algae is based on adsorption of the alkylammonium cation on the bacterial cell surface, diffusion through the cell wall and then binding and disruption of cytoplasmatic membrane. Damage to the membrane results in a release of potassium ions and other cytoplasmatic constituents, finally leading to the death of the cell [14,15,16,17]. 

However, in recent years there have been frequent reports of cases of increasing resistance of certain microorganism strains to the biocidal activity of benzalkonium chlorides [18,19,20,21]. The use of inappropriate concentrations of benzalkonium chlorides and incomplete removal of residues of these microbiocides after finishing the disinfection process exposes microorganisms to prolonged contact with sublethal doses of BACs. This contributes to the development of defensive mechanisms and the emergence of strains of microorganisms exhibiting resistance to benzalkonium chlorides as well as cross-resistance to certain other antibiotics [22,23]. This fact creates a serious epidemiological threat, especially in medical facilities, manifested by an increase in morbidity and mortality among patients [24]. One of the way to overcome this serious negative side effect is a periodically application of new microbiocides with modified structures.

To meet this challenge we have proposed a modification of benzalkonium chlorides based on the introduction of nitrogen atoms into the aromatic ring. For that reason a series of novel quaternary ammonium compounds **P13**–**P18** with variable alkyl chain length and bearing pyridine rings instead of phenyl rings were synthesized. In the literature there are known nonionic surfactants containing 4-pyridyl moieties, for example amines [25,26,27], amides [28], ethers and ketones [29,30], as well as 4-alkylpyridines [31]. According to our knowledge this is the first report however about the synthesis of a series of benzalkonium chloride analogues bearing 4-pyridyl rings.

## 2. Results and Discussion

**Scheme 1:**
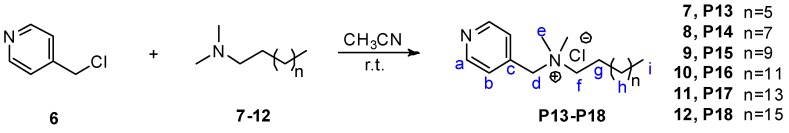
Synthesis of compounds **P13**–**P18**.

The novel compounds **P13**–**P18** were obtained according to Scheme 1 by Menshutkin reaction of 4-chloromethylpyridine (**6**) with the corresponding *N*,*N*-dimethylalkylamines **7**–**12** containing 8, 10, 12, 14, 16, 18 carbon atoms in alkyl chain, respectively. The use of the polar aprotic solvent acetonitrile as a reaction medium allowed us to obtain compounds **P13**–**P18** in good yield at room temperature. 

Crude products were purified by crystallization from 1:1 acetonitrile/acetone mixtures and characterized by spectroscopic methods. Copies of recorded spectra of compounds **P13**–**P18** are available in Supplementary Materials.

### 2.1. Spectroscopic Characterization

#### 2.1.1. Nuclear Magnetic Resonance Spectra

The most characteristic feature of the ^1^H-NMR spectra of the title compounds are the signals of the pyridine protons and the protons of the methyl and methylene groups directly linked to the positively charged nitrogen atom. The chemical shifts of the H-a and H-b protons of the pyridine ring are observed around 8.70–8.71 and 7.72–7.73 ppm, respectively. They are shifted toward the higher ppm values than those observed for γ-picoline [32], for example. This is caused by the proximity of the positively charged nitrogen atom linked via methylene groups to the C-c carbon atom of the pyridine ring (Scheme 1). Protons H-d and H-e of then methylene and methyl groups directly attached to the quaternary nitrogen atom appear in ^1^H-NMR spectra as singlets in the 5.30–5.24 ppm and 3.54–3.52 ppm regions, respectively. In all ^1^H-NMR spectra of the analyzed surfactants **P13**–**P18** H_2_O proton signals are observed in the range 2.98–2.65 ppm. According to literature data the protons of H_2_O in CDCl_3_ resonate at 1.56 ppm [33]. The observed shifts over 1 ppm toward the higher ppm values in compounds **P13**–**P18** spectra indicate the presence of weak hydrogen bonding between the water molecules and chloride anions.

The ^13^C-NMR spectra of **P13**–**P18** show three characteristic groups of signals in the range of 150.65–150.70, 127.58–127.59 and 136.11–136.15 ppm, which are assigned to C-a, C-b and C-c carbon atoms of the pyridine rings, respectively. The carbon atoms of the methylene groups C-d and C-f resonate at 65.58–65.49 ppm and 64.07–64.02 ppm, respectively, whereas the carbon atoms of the methyl group C-e are observed at 49.88–49.79 ppm.

#### 2.1.2. Infrared Spectra

In the FT-IR spectra of **P13**–**P18** some a very characteristic bands are observed. The stretching vibrations bands of pyridine C–H bonds are observed in the range 3062–3005 cm^−1^ while deformational vibrations, in plane and out of plane, lie in the 1351–1069 cm^−1^ and 829–820 cm^−1^ regions, respectively. Stretching vibrations bands of aromatic ring C=C and C=N bonds are observed in a typical 1617–1562 cm^−1^ region. Deformational and skeletal vibrations bands of the pyridine rings are observed in the 1419–1415 cm^−1^ and 1006–996 cm^−1^ regions, respectively. The symmetric and asymmetric stretching vibrations of C–H bonds of methyl and methylene groups lie in the 2985–2850 cm^−1^ region. Bands observed in the 3454–3242 cm^−1^ region are assigned to stretching vibrations of hydrogen bonded H_2_O molecules.

#### 2.1.3. ESI-MS

In ESI-MS in positive ion mode of all analyzed surfactants the presence of the corresponding [M^+^] ion was observed as the strongest peak. The presence of a [2M + Cl]^+^ ion peak was also observed. For all discussed compounds in negative ion mode the presence of a [M + 2Cl]^−^ ion was observed. Taken together, the ESI-MS spectra, as well as ^1^H-NMR, ^13^C-NMR and FT-IR spectra confirmed the structure and purity of the obtained compounds.

### 2.2. Semiempirical Calculations

**Figure 2:**
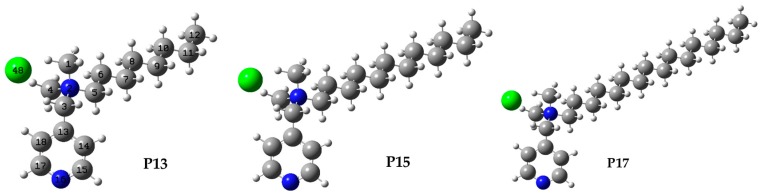
Representative molecular models of compounds **P13**, **P15** and **P17**.

PM3 semiempirical calculations were performed using the Gaussian03W program. Some representative molecular models for **P13**, **P15** and **P17** are shown in Figure 2. The heats of formation (HOF) and geometric parameters of compounds **P13**–**P18** are presented in Table 1. The spatial arrangement of atoms around the tetrahedral quaternary nitrogen atom as well as the geometry of the alkyl chains of compounds **P13**–**P18** are very similar to those of other alkyl ammonium salts [34]. The change from phenyl ring to pyridine ring has a little effect on the geometric parameters. Similarly the nature of the anion, bromide or chloride, has practically no effect on the bond lengths and angles in alkylammonium salts. What is more, the calculated parameters in gas phase correspond to some extent to the parameters of the molecule geometry in crystal form (Table 1).

**Figure 3:**
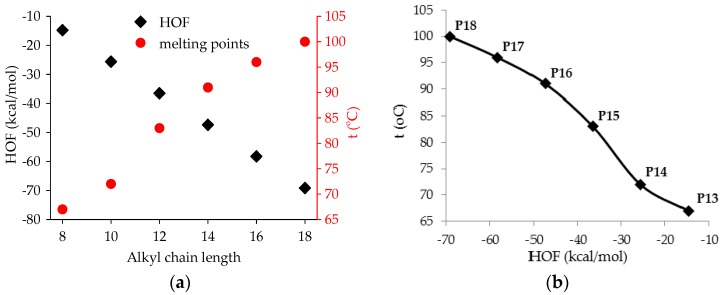
Plot (**a**) The relationships between calculated HOF and measured melting points vs. the length of the hydrocarbon chain of compounds **P13**–**P18**; Plot (**b**) The correlation of melting points vs. HOF of **P13**–**P18**.

This allows one to extrapolate some conclusions from the calculated structure to the condensed phase. The heat of formation (HOF) of the synthesized compounds decreases linearly with an increasing number of methylene groups in the hydrocarbon chain (Figure 3a). The higher the number of methylene units, the lower the heat of formation. There is also a good correlation between the melting points of *N*,*N*-dimethyl-*N*-(4-methylpyridyl)-*N*-alkylammonium chlorides and the number of methylene groups in their alkyl chains (Figure 3a). The higher melting points reflect the better packing in the crystal what is a result of the strong hydrophobic interactions between straight hydrocarbon chains. An observed increasing stability of *N*,*N*-dimethyl-*N*-(4-methylpyridyl)-*N*-alkylammonium chlorides with the lengthening of the alkyl chain is in a good accordance with the increasing melting points of these compounds, which reflect a sigmoidal type of correlation (Figure 3b).

### 2.3. Aggregation Behavior

The aggregation behavior of the synthesized surfactants was evaluated by a conductometric study performed in aqueous solutions at 25 °C. Critical micellization concentrations (CMCs) for compounds **P14**–**P18** were obtained from the plots of specific conductivity (κ) against the concentrations of the surfactants. A common method of estimation of CMC values from conductivity vs. surfactant concentration plots is by finding the intersection point of two least squares method fitted lines assigned for pre- and post-micellar regions. Unfortunately, this method is not suitable for all types of surfactants, as in some cases an inflection point is very difficult to find because of the low curvature of the plots. In such cases, the measurements are subject to large errors. In the case of the synthesized compounds, their CMCs were difficult to estimate by the conventional method. For that reason critical micellization concentrations for compounds **P14**–**P18** were obtained by the analysis of the plots of differential conductivity (dκ/dc) vs. surfactant concentration. This is more precise approach that has been successfully applied in determining CMC values [36].

The plots of dκ/dc vs. concentration possess reverse sigmoid shapes and thus can be fitted to a Boltzmann-type sigmoid expressed by the following equation:

(1) $$y=A_{2}+\;\frac{A_{1}-\;A_{2}}{1+e^{\frac{x-x_{0}}{dx}}}$$

**Figure 4:**
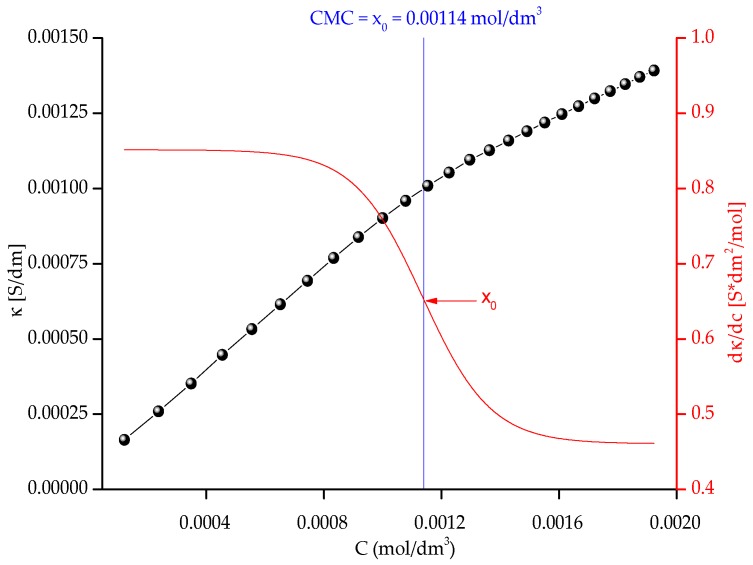
Specific conductivity (•) and differential conductivity Boltzman-type fitting (―) vs. concentration of **P17** at 25 °C.

Parameters A1 and A2 represent the pre-micellar and post-micellar slopes, respectively. The width of transition, *dx*, possesses a central point, *x*_0_, corresponding to the CMC value (Figure 4). CMC values for compounds **P14**–**P18** are given in Table 2. **P14**–**P17** display CMC values typical of cationic surfactants that decrease significantly with increasing alkyl chain length, because of the increase of the hydrophobic character of the surfactants. In the case of **P18** the increase of alkyl chain length causes a less significant change in CMC values probably because of coiling of the long alkyl chains in water [37].

**Figure 5:**
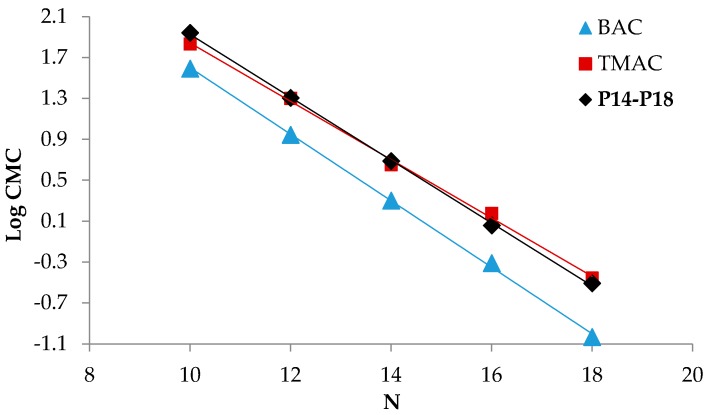
Graphical presentation of the Klevens equation for **P14**–**P18**, TMAC and BAC (N = alkyl chain length).

The novel compounds possess CMCs much higher than benzalkonium chlorides bearing alkyl chains of the same length. The data obtained from Figure 5 and Table 2 indicate that the compounds presented in this paper exhibit micellization behavior more similar to that of trimethylalkylammonium chlorides rather than to BACs. The Klevens equation (2) is often used to characterize the relation between the CMC of a surfactant and its structure [41]. It presents a linear relationship between the log CMC and alkyl chain length of the surfactant:

(2) logCMC=A−BN

where A is a constant that reflects the free energy change related to transfer of hydrophilic part of the surfactant from the aqueous phase to the micelle, whereas parameter B determines the free energy change associated with the transfer of a methylene (−CH_2_−) unit of the hydrophobic chain from aqueous solution into the micelle. N is the number of carbon atoms in alkyl chain of surfactant.

The value of B obtained from linear fitting of the Klevens equation (Table 2) allowed us to determine the change of the Gibbs free energy of methylene group transfer, ΔG_(−CH2−)_, according to the equation:

(3) $${\Delta G}_{\left(-\mathrm{CH}2-\right)}=-2.3\mathrm{RTB}$$

where R is the universal gas constant and T is the absolute temperature. The Gibbs free energy of methylene group transfer equals −1.71 kJ/mol, −1.63 kJ/mol and −1.85 kJ/mol for **P14**–**P18**, trimethylalkylammonium chlorides and benzalkonium chlorides, respectively. The lower the Gibbs free energy ΔG_(−CH2−)_ the easier the process of micellization. Alkyl chain transfer from the aqueous phase into the interior of the micelle reduces the energy of the solution, which promotes micellization. However, within the micelles electrostatic repulsion exists between the positively charged molecules of the surfactants, which hinders the micellization. Consequently, the CMC value depends on the balance between the factors supporting and counteracting micellization. Therefore the hydrophilic head structure it is an important factor determining the phenomenon of micellization. Benzalkonium chlorides having within their hydrophilic part benzene rings lowering the polarity of the whole molecule, possess much lower CMC values than trimethylalkylammonium chlorides. The newly prepared salts have a pyridine ring that possesses a dipole moment and is incorporated into their hydrophilic part. The presence of an electronegative nitrogen atom having a lone pair of electrons may contribute to the additional repulsive interactions of the hydrophilic part of the surfactant, resulting in increased CMC values as compared with the benzalkonium compounds.

### 2.4. Antimicrobial Properties

Minimal inhibitory concentrations (MIC) of *N*,*N*-dimethyl-*N*-(4-methylpyridyl)-*N*-alkyl-ammonium chlorides have been determined against microscopic fungi, *A. niger*, *C. albicans* and *P. chrysogenum* as well as bacteria, *S. aureus, B. subtilis, E. coli* and *P. aeruginosa* (Table 3). Since the octadecyl derivative of the title compounds was not sufficiently soluble in water, therefore only the octyl, decyl, dodecyl, tetradecyl and hexadecyl derivatives were tested.

**Figure 6:**
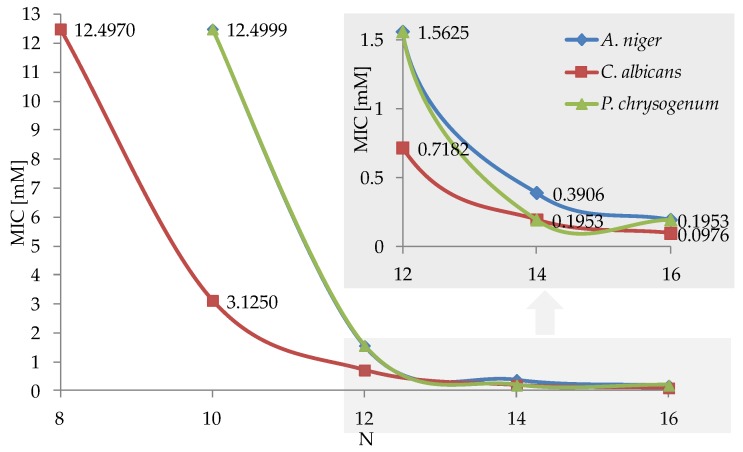
Antifungal activity of **P13**–**P17** (N = alkyl chain length).

**Figure 7:**
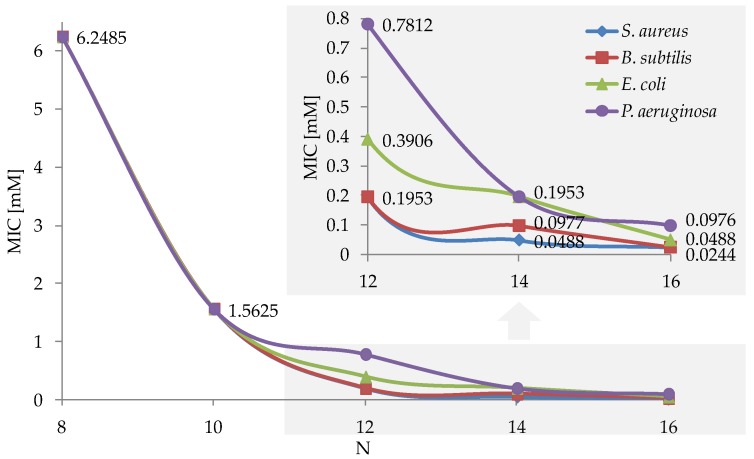
Antibacterial activity of **P13**–**P17** (N = alkyl chain length).

The MIC values of *N*,*N*-dimethyl-*N*-(4-methylpyridyl)-*N*-alkylammonium chlorides for fungi range from 0.1 to 12 mM. The highest MIC values are obtained for the derivatives with the shortest alkyl chains. As the length of the hydrocarbon chain increases the MIC in general decreases (Figure 6). The lowest MIC values, i.e., the highest antifungal activity, are observed for the hexadecyl derivatives. *Aspergillus niger* is usually more resistant to chemical disinfectants than other microscopic fungi and is very difficult to eradicate from any medical or food industry environment. The MIC values of *N*,*N*-dimethyl-*N*-(4-methylpyridyl)-*N*-alkylammonium chlorides for all tested fungi are similar, what means that decontamination of any area can be done using optimized concentrations of *N*,*N*-dimethyl-*N*-(4-methylpyridyl)-*N*-alkylammonium chlorides without the need to use a high concentration of microbiocides to effectively remove of *Aspergillus niger*. MIC values of pyridyl analogues of BAC for the bacteria *S. aureus, B. subtilis*, *E. coli* and *P. aeruginosa* are lower than those for microscopic fungi and range from 6 to 0.02 mM (Table 3). Similarly to the effects observed for microscopic fungi, the MIC values of alkyl derivatives for bacteria decrease with the increasing length of the hydrocarbon chain (Figure 7). The antimicrobial activity of alkylammonium salts is generally dependant on the length of hydrocarbon chain what is connected with the potential to penetrate the cell wall. For benzalkonium chlorides and similar compounds the lowest MIC values are observed for dodecyl and tetradecyl derivatives (Table 4). The shorter hydrocarbon chain is not able to penetrate the cell wall; the longer substituent is too flexible that is why it cannot also infiltrate the cell wall. The relationships between MIC and the number of carbon atoms in the alkyl substituent are usually of parabolic type, with minima around C12-C14. For *N*,*N*-dimethyl-*N*-(4-methylpyridyl)-*N*-alkylammonium chlorides the lowest MIC values are observed for C16, i.e., at a slightly higher chain length than for benzalkonium chlorides. Because of the low solubility of longer alkyl derivatives we were unable to check their MIC and compare it with BACs. In general benzalkonium chlorides show slightly lower MIC values in comparison to the pyridyl analogues, however for *Pseudomonas aeruginosa* the C16 pyridyl analogue showed better antimicrobial activity (Table 4).

## 3. Materials and Methods

### 3.1. General Information

The NMR spectra were measured in CDCl_3_ as a solvent with a 300 MHz Mercury NMR spectrometer (Varian, Oxford, UK). Infrared spectra of **P13**, **P15**, **P16** and **P18** were recorded as KBr pellets using a IFS 66 FT-IR spectrometer (Bruker, Karlsruhe, Germany) at 295 K. Infrared spectra of **P14** and **P17** were recorded on a ATR 4700 type FT-IR spectrometer (Jasco, Easton, MD, USA). The Electron Spray Ionization (ESI) mass spectra were recorded in methanol on a ZQ mass spectrometer (Waters/Micromass, Manchester, UK) equipped with a syringe pump (Harvard Apparatus, St. Laurent, QC, Canada). *N*,*N*-dimethylamines were purchased from Aldrich (Poznań, Poland) except for *N*,*N*-dimethyloctadecylamine that was purchased from TCI (Łódź, Poland). 4-Chloromethylpyridine (**6**) was obtained from 4-chloromethylpyridine hydrochloride (purchased from Alfa Aesar, Karlsruhe, Germany) according to a literature procedure [43] and used further without purification. Organic solvents were purchased from POCH (Gliwice, Poland) except for acetonitrile that was purchased from VWR (Gdańsk, Poland).

### 3.2. Typical Procedure of Synthesis of N,N-Dimethyl-N-(4-methylpyridyl)-N-alkylammonium Chlorides ***P13**–**P18***

A mixture of 4-chloromethylpyridine (**6**, 1 g, 7.87 mmol) and an *N,N*-dimethylalkylamine (7.87 mmol) in CH_3_CN (10 mL) was stirred at room temperature for 24 h. Completion of reaction was controlled by TLC (CHCl_3_:MeOH 5:1). Then the solvent was evaporated under reduced pressure and the residue was washed twice with diethyl ether. The crude dark brown product was purified by recrystallization from acetone/acetonitrile (1:1).

*N,N-Dimethyl-N-(4-methylpyridyl)-N-octylammonium chloride* (**P13**). Light brown solid, yield: 79%; m.p. 67–70 °C; IR (KBr) ν_max_ 3057, 2929, 2850, 1596, 1473 cm^−1^; ^1^H-NMR (CDCl_3_) δ 8.71 (2 H, d, *J* = 5.9 Hz, H-a), 7.73 (2 H, d, *J* = 6.0 Hz, H-b), 5.24 (2 H, s, H-d), 3.53 (2 H, m, H-f), 3.34 (6 H, s, H-e), 1.80 (2 H, m, H-g), 1.15–1.39 (10 H, m, H-h), 0.87 (3 H, t, *J* = 6.6, 6.9 Hz, H-i); ^13^C-NMR (CDCl_3_) δ 150.65 (CH, C-a), 136.17 (C, C-c), 127.59 (CH, C-b), 65.58 (CH_2_, C-d), 64.06 (CH_2_, C-f), 49.86 (CH_3_, C-e), 31.47 (CH_2_, C-g), 29.05–22.43 (CH_2_, C-h), 13.92 (CH, C-d); ESI-MS *m*/*z*: 249 [M]^+^.

*N,N-Dimethyl-N-(4-methylpyridyl)-N-decylammonium chloride* (**P14**). Light orange solid, yield: 76%; m.p. 72–75 °C; IR (KBr) ν_max_ 3023, 2921, 2850, 1601, 1470 cm^−1^; ^1^H-NMR (CDCl_3_) δ 8.71 (2 H, d, *J* = 5.9 Hz, H-a), 7.72 (2 H, d, *J* = 6.0 Hz, H-b), 5.26 (2 H, s, H-d), 3.53 (2 H, m, H-f), 3.34 (6 H, s, H-e), 1.80 (2 H, m, H-g), 1.15–1.39 (10 H, m, H-h), 0.88 (3 H, t, *J* = 6.6, 6.9 Hz, H-i); ^13^C-NMR (CDCl_3_) δ 150.67 (CH, C-a), 136.14 (C, C-c), 127.59 (CH, C-b), 65.57 (CH_2_, C-d), 64.04 (CH_2_, C-f), 49.86 (CH_3_, C-e), 31.71 (CH_2_, C-g), 29.27–22.53 (CH_2_, C-h), 14.00 (CH_3_, C-i); ESI-MS *m*/*z*: 277 [M]^+^.

*N,N-Dimethyl-N-(4-methylpyridyl)-N-dodecylammonium chloride* (**P15**). Light brown solid, yield: 81%; m.p. 83–86 °C; IR (KBr) ν_max_ 3062, 2920, 2850, 1602, 1470 cm^-1^; ^1^H-NMR (CDCl_3_) δ 8.70 (2 H, d, *J* = 5.9 Hz, H-a), 7.72 (2 H, d, *J* = 6.0 Hz, H-b), 5.25 (2 H, s, H-d), 3.52 (2 H, m, H-f), 3.34 (6 H, s, H-e), 1.80 (2 H, m, H-g), 1.15–1.39 (10 H, m, H-h), 0.88 (3 H, t, *J* = 6.6, 6.9 Hz, H-i); ^13^C-NMR (CDCl_3_) δ 150.66 (CH, C-a), 136.14 (C, C-c), 127.58 (CH, C-b), 65.57 (CH2, C-d), 64.02 (CH2, C-f), 49.86 (CH3, C-e), 31.77 (CH2, C-g), 29.46–22.56 (CH_2_, C-h), 14.00 (CH3, C-i); ESI-MS *m*/*z*: 305 [M]^+^.

*N,N-Dimethyl-N-(4-methylpyridyl)-N-tetradecylammonium chloride* (**P16**). Light brown solid, yield: 83%; m.p. 91–93 °C; IR (KBr) ν_max_ 3062, 2915, 2850, 1602, 1470 cm^-1^; ^1^H-NMR (CDCl_3_) δ 8.71 (2 H, d, *J* = 5.9 Hz, H-a), 7.73 (2 H, d, *J* = 6.0 Hz, H-b), 5.30 (2 H, s, H-d), 3.54 (2 H, m, H-f), 3.35 (6 H, s, H-e), 1.80 (2 H, m, H-g), 1.20–1.39 (10 H, m, H-h), 0.88 (3 H, t, *J* = 6.8, 7.3 Hz, H-i); ^13^C-NMR (CDCl_3_) δ 150.68 (CH, C-a), 136.12 (C, C-c), 127.58 (CH, C-b), 65.49 (CH2, C-d), 64.02 (CH2, C-f), 49.79 (CH3, C-e), 31.80 (CH2, C-g), 29.53–22.58 (CH2, C-h), 14.03 (CH3, C-i); ESI-MS *m*/*z*: 333 [M]^+^.

*N,N-Dimethyl-N-(4-methylpyridyl)-N-hexadecylammonium chloride* (**P17**). Light brown solid, yield: 85%; m.p. 96–98 °C; IR (KBr) ν_max_ 3053, 2921, 2850, 1601, 1470 cm^-1^; ^1^H-NMR (CDCl_3_) δ 8.71 (2 H, d, *J* = 5.9 Hz, H-a), 7.73 (2 H, d, *J* = 6.0 Hz, H-b), 5.30 (2 H, s, H-d), 3.54 (2 H, m, H-f), 3.35 (6 H, s, H-e), 1.80 (2 H, m, H-g), 1.19–1.37 (10 H, m, H-h), 0.88 (3 H, t, *J* = 6.6, 6.9 Hz, H-i); ^13^C-NMR (CDCl_3_) δ 150.70 (CH, C-a), 136.11 (C, C-c), 127.58 (CH, C-b), 65.51 (CH2, C-d), 64.04 (CH2, C-f), 49.80 (CH3, C-e), 31.83 (CH2, C-g), 29.61–22.59 (CH2, C-h), 14.04 (CH3, C-i); ESI-MS *m*/*z*: 361 [M]+.

*N,N-Dimethyl-N-(4-methylpyridyl)-N-octadecylammonium chloride* (**P18**). Light brown solid, 88%; m.p. 100–102 °C; IR (KBr) ν_max_ 3054, 2915, 2851, 1600, 1472 cm^-1^; ^1^H-NMR (CDCl_3_) δ 8.71 (2 H, d, *J* = 5.9 Hz, H-a), 7.73 (2 H, d, *J* = 6.0 Hz, H-b), 5.30 (2 H, s, H-d), 3.54 (2 H, m, H-f), 3.35 (6 H, s, H-e), 1.80 (2 H, m, H-g), 1.17–1.35 (30 H, m, H-h), 0.87 (3 H, t, *J* = 6.4, 7.3 Hz, H-i); ^13^C-NMR (CDCl_3_) δ 150.68 (CH, C-a), 136.12 (C, C-c), 127.58 (CH, C-b), 65.49 (CH2, C-d), 64.02 (CH2, C-f), 49.79 (CH3, C-e), 31.80 (CH2, C-g), 29.56–22.58 (CH2, C-h), 14.03 (CH3, C-i); ESI-MS *m*/*z*: 389 [M]+.

### 3.3. Antimicrobial Properties Evaluation

The MIC values of **P13**–**P17** against bacteria and microscopic fungi (yeast and moulds) were measured at the Institute of Fermentation Technology and Microbiology, Technical University of Lodz in Poland. The MICs are defined as the lowest concentration of the compounds at which there was no visible growth of microorganisms. MIC values were determined by a standard tube 2-fold dilution method [44,45]. The experiments were carried out on the bacteria *Escherichia coli* ATCC 10536, *Pseudomonas aeruginosa* ATCC 85327, *Staphylococcus aureus* ATCC 6538, *Bacillus subtilis* NCAM 01644 and microscopic fungi *Candida albicans* ATCC 10231, *Aspergillus niger* ATCC 16404, *Penicillium chrysogenum* ATCC 60739. Antifungal properties were checked on a Malt Extract Broth medium (Merck, Darmstadt, Germany) at a density of inoculum of 1–2 × 10^6^ cfu/mL; antibacterial activity—on Trypticase Soy Broth (Merck), density of inoculum 1–2 × 10^7^ cfu/mL. All tests were repeated three times.

### 3.4. Conductometric Study

Measurements of conductivity were obtaining using a CO300 conductivity meter (VWR, Gdańsk, Poland). CMC values of compounds **P14**–**P18** were obtained from conductometric titrations conducted in double distilled water in 25 °C. For **P14**–**P16,** to an initial volume of surfactant solution water was added gradually, and after every addition the conductivity of the solution was measured. For **P17** and **P18** to an initial volume of water surfactant solution was added gradually, and after every addition conductivity of the solution was measured. For each compound, analysis was conducted twice and CMC values reported in this work are the average of the two results.

## Figures and Tables

**Table 1:** HOF, bond lengths and angles for **P13**–**P18**, BDDAC and BDDAB.

	Compound								
	P13	P14	P15	P16	P17	P18	BDDAC ^1^	BDDAB ^2^	BDDAB ^3^
							(Calculated)	(Calculated)	(Crystal)
**Heat of Formation [kcal/mol]**	−14.766	−25.638	−36.512	−47.387	−58.264	−69.141	−44.786	−37.285	-
**Distance [Å]**									
N(2)^...^X^−^	3.137	3.137	3.137	3.137	3.137	3.137	3.148	3.535	4.301
N(2)–C(1)	1.508	1.508	1.508	1.508	1.508	1.508	1.508	1.515	1.517
N(2)–C(4)	1.502	1.502	1.502	1.502	1.502	1.502	1.502	1.510	1.531
N(2)–C(3)	1.536	1.536	1.536	1.536	1.536	1.536	1.537	1.539	1.546
N(2)–C(5)	1.529	1.529	1.529	1.529	1.529	1.529	1.528	1.534	1.510
C(5)–C(6)	1.525	1.525	1.525	1.525	1.525	1.525	1.525	1.526	1.538
C(3)–C(13)	1.494	1.494	1.494	1.494	1.494	1.494	1.494	1.492	1.487
C(13)–C(14)	1.397	1.397	1.397	1.397	1.397	1.397	1.397	1.397	1.402
C(14)–C(15)	1.394	1.394	1.394	1.394	1.394	1.394	1.390	1.389	1.393
C(15)–N(16)	1.353	1.353	1.353	1.353	1.353	1.353	-	-	-
N(16)–C(17)	1.352	1.352	1.352	1.352	1.352	1.352	-	-	-
C(17)–C(18)	1.397	1.397	1.397	1.397	1.397	1.397	1.391	1.392	1.356
C(13)–C(18)	1.396	1.396	1.396	1.396	1.396	1.396	1.397	1.398	1.363
**Angle [°]**									
C(1)–N(2)–C(4)	108.556	108.558	108.558	108.560	108.558	108.558	108.556	109.668	109.560
C(1)–N(2)–C(3)	107.159	107.159	107.157	107.158	107.158	107.159	107.223	108.079	108.440
C(1)–N(2)–C(5)	110.034	110.035	110.034	110.034	110.035	110.035	109.989	108.868	110.100
C(3)–N(2)–C(4)	110.028	110.024	110.024	110.024	110.024	110.023	109.982	112.142	108.160
C(3)–N(2)–C(5)	110.391	110.393	110.395	110.392	110.393	110.393	110.446	109.140	112.530
C(4)–N(2)–C(5)	110.593	110.593	110.594	110.593	110.594	110.593	110.566	108.890	107.990
N(2)–C(3)–C(13)	114.561	114.562	114.564	114.562	114.562	114.561	114.413	113.849	116.070
N(2)–C(5)–C(6)	112.170	112.171	112.171	112.171	112.171	112.170	112.243	112.948	116.240
**Dihedral [°]**									
N(2)–C(3)–C(13)–C(14)	90.143	90.145	90.140	90.145	90.139	90.146	90.138	89.914	89.500
N(2)–C(3)–C(13)–C(18)	−93.233	−93.232	−93.240	−93.233	−93.241	−93.233	−93.370	−93.929	−89.540
C(4)–N(2)–C(3)–C(13)	58.616	58.621	58.630	58.620	58.632	58.626	60.176	44.503	60.950
C(5)–N(2)–C(3)–C(13)	−63.720	−63.715	−63.707	−63.715	−63.704	−63.709	−62.133	−76.241	−58.260
C(1)–N(2)–C(3)–C(13)	176.460	176.463	176.471	176.465	176.474	176.469	178.030	165.504	179.690

^1^ benzyldimethyldodecylammonium chloride; ^2^ benzyldimethyldodecylammonium bromide; ^3^ Ref. [35].

**Table 2:** CMC values and Klevens equation parameters of **P14**–**P18**, benzalkonium chlorides (BAC) and trimethylammonium chlorides (TMAC).

	CMC [mM]					Klevens Equation Parameters		
N ^1^	10	12	14	16	18	A	B	R ^2^
**P14**–**P18**	87.12	20.17	4.87	1.14	0.310	1.9978	0.3073	0.9996
**TMAC**	68.0 ^2^	20.0 ^2^	4.50 ^2^	1.50 ^3^	0.35 ^3^	1.6927	0.2851	0.9983
**BAC**	39.0 ^2^	8.8 ^2^	2.00 ^2^	0.49 ^4^	0.093 ^5^	1.8487	0.325	0.9994

^1^ **N** = alkyl chain length; ^2^ Ref. [37]; ^3^ Ref. [38]; ^4^ Ref. [39]; ^5^ Ref. [40].

**Table 3:** Antimicrobial properties of **P13**–**P17**.

Compound	MIC [mM]						
	*A. niger*	*C. albicans*	*P. chrysogenum*	*S. aureus*	*B. subtilis*	*E. coli*	*P. aeruginosa*
**P13**	-	12.497	-	6.2485	6.2485	6.2485	6.2485
**P14**	12.499	3.125	12.4999	1.5625	1.5625	1.5625	1.5625
**P15**	1.5625	0.7182	1.5625	0.1953	0.1953	0.3906	0.7812
**P16**	0.3906	0.1953	0.1953	0.0488	0.0977	0.1953	0.1953
**P17**	0.1953	0.0976	0.1953	0.0244	0.0244	0.0488	0.09764

**Table 4:** Literature values of MIC of benzalkonium chlorides [42].

N ^1^	MIC [mM]		
	*P. aeruginosa*	*A. niger*	*C. albicans*
**8**	5	5	2.5
**10**	1.25	0.625	0.312
**12**	0.312	0.0781	0.039
**14**	0.0781	0.0195	0.0097
**16**	0.156	0.156	0.0195

^1^ N = alkyl chain length.

## Supplementary Materials

Supplementary materials can be accessed at: http://www.mdpi.com/1420-3049/22/1/130/s1.

## References

[1] Espinoza R. (2004). Multivesicular Emulsion Drug Delivery Systems. U.S. Patent.

[2] Goredema A., Strong A., Odell P., Allen C.G., Birau M.M. (2012). Low Molecular Weight Quaternary Ammonium Salt Dispersants. U.S. Patent.

[3] Yamada H., Urata C., Higashitamori S., Aoyama Y., Yamauchi Y., Kuroda K. (2014). Critical roles of cationic surfactants in the preparation of colloidal mesostructured silica nanoparticles: Control of mesostructure, particle size, and dispersion. ACS Appl. Mater. Interfaces.

[4] Igarashi T., Morita N., Okamoto Y., Nakamura K. (2015). Elucidation of softening mechanism in rinse cycle fabric softeners. Part 1: Effect of hydrogen bonding. J. Surfactants Deterg..

[5] Okawa H., Hanabusa K., Suzuki M., Fukui H., Sakurai S. (2014). Novel gemini compounds bearing an amide group show water solubility and useful functions as cosmetic ingredients. Int. J. Res. Cosmet. Sci..

[6] Jones R.A. (2000). Quaternary Ammonium Salts: Their Use in Phase-Transfer Catalysis.

[7] Denmark S.E., Gould N.D., Wolf L.M. (2011). A systematic investigation of quaternary ammonium ions as asymmetric phase-transfer catalysts. Application of quantitative structure activity/selectivity relationships. J. Org. Chem..

[8] Zhi D., Zhang S., Cui S., Zhao Y., Wang Y., Zhao D. (2013). The headgroup evolution of cationic lipids for gene delivery. Bioconjug. Chem..

[9] Koziróg A., Rajkowska K., Otlewska A., Piotrowska M., Kunicka-Styczyńska A., Brycki B., Nowicka-Krawczyk P., Kościelniak M., Gutarowska B. (2016). Protection of historical wood against microbial degradation—Selection and application of microbiocides. Int. J. Mol. Sci..

[10] Jennings M.C., Minbiole K.P.C., Wuest W.M. (2015). Quaternary ammonium compounds: An antimicrobial mainstay and platform for innovation to address bacterial resistance. ACS Infect. Dis..

[11] Niu L., Zhang H., Wei F., Wu S., Cao X., Liu P. (2005). Corrosion inhibition of iron in acidic solutions by alkyl quaternary ammonium halides: Correlation between inhibition efficiency and molecular structure. Appl. Surf. Sci..

[12] Henry K.M., Hicks K.D. (2015). Bis-Quaternary Ammonium Salt Corrosion Inhibitors. U.S. Patent.

[13] Domagk G. (1935). Eine neue Klasse von Desinfektionsmitteln. DMW Dtsch. Med. Wochenschr..

[14] Block S.S. (2001). Disinfection, Sterilization, and Preservation.

[15] Brycki B. (2010). Gemini alkylammonium salts as biodeterioration inhibitors. Pol. J. Microbiol..

[16] Fraise A.P., Lambert P.A., Maillard J.-Y. (2008). Russell, Hugo & Ayliffe’s Principles and Practice of Disinfection, Preservation & Sterilization.

[17] Paulson D.S. (2002). Handbook of Topical Antimicrobials: Industrial Applications in Consumer Products and Pharmaceuticals.

[18] Keen P.L., Montforts M.H.M.M. (2012). Antimicrobial Resistance in the Environment.

[19] Ascenzi J.M. (1995). Handbook of Disinfectants and Antiseptics.

[20] McDonnell G.E. (2013). Antisepsis, Disinfection, and Sterilization: Types, Action, and Resistance.

[21] Tezel U., Pavlostathis S.G. (2015). Quaternary ammonium disinfectants: Microbial adaptation, degradation and ecology. Curr. Opin. Biotechnol..

[22] Mc Cay P.H., Ocampo-Sosa A.A., Fleming G.T.A. (2010). Effect of subinhibitory concentrations of benzalkonium chloride on the competitiveness of *Pseudomonas aeruginosa* grown in continuous culture. Microbiology.

[23] Tandukar M., Oh S., Tezel U., Konstantinidis K.T., Pavlostathis S.G. (2013). Long-term exposure to benzalkonium chloride disinfectants results in change of microbial community structure and increased antimicrobial resistance. Environ. Sci. Technol..

[24] Levy S.B., Marshall B. (2004). Antibacterial resistance worldwide: Causes, challenges and responses. Nat. Med..

[25] Bandyopadhyay P., Jha S., Imran Ali S.K. (2009). Picolyl alkyl amines as novel tyrosinase inhibitors: Influence of hydrophobicity and substitution. J. Agric. Food Chem..

[26] Diaz-Fernandez Y., Foti F., Mangano C., Pallavicini P., Patroni S., Perez-Gramatges A., Rodriguez-Calvo S. (2006). Micelles for the self-assembly of “off-on-off” fluorescent sensors for pH windows. Chemistry.

[27] Tripathi R.P., Saxena N., Tiwari V.K., Verma S.S., Chaturvedi V., Manju Y.K., Srivastva A.K., Gaikwad A., Sinha S. (2006). Synthesis and antitubercular activity of substituted phenylmethyl- and pyridylmethyl-amines. Bioorg. Med. Chem..

[28] Sagara T., Uematsu K., Nagata K. (2003). Dynamic phase change and adsorption/desorption of 4-pyridyl terminated amphiphiles possessing an amide functionality at a Au(111) electrode as tracked by electrochemical measurements. J. Electroanal. Chem..

[29] Uematsu K., Sagara T. (2008). Voltammetric study of adsorption layers of various 4-pyridyl terminated surfactants on a Au(111) electrode: Effects of electronic property of pyridyl group and intermolecular hydrogen bonding upon potential-driven phase changes. J. Electroanal. Chem..

[30] Uematsu K., Sagara T. (2008). Voltammetric study of insoluble 4-pyridyl-terminated surfactant binary-component films prepared by multiple horizontal touching method on a Au(1 1 1) electrode. Colloids Surf. Physicochem. Eng. Asp..

[31] Oertling H. (2010). 4-Alkyl Substituted Pyridines as Odiferous Substances. U.S. Patent.

[32] Pretsch E., Bühlmann P., Badertscher M. (2009). Structure Determination of Organic Compounds: Tables of Spectral Data.

[33] Gottlieb H.E., Kotlyar V., Nudelman A. (1997). NMR Chemical shifts of common laboratory solvents as trace impurities. J. Org. Chem..

[34] Lundén B.M. (1974). The crystal structure of *n*-dodecylammonium bromide. Acta Crystallogr. B.

[35] Rodier N., Dugué J., Céolin R., Baziard-Mouysset G., Stigliani J.L., Payard M. (1995). Bromure de benzododécinium monohydraté. Acta Crystallogr. C.

[36] Mata J., Varade D., Bahadur P. (2005). Aggregation behavior of quaternary salt based cationic surfactants. Thermochim. Acta.

[37] Rosen M.J., Kunjappu J.T. (2012). Surfactants and Interfacial Phenomena.

[38] Cella J.A., Eggenberger D.N., Noel D.R., Harriman L.A., Harwood H.J. (1952). The relation of structure and critical concentration to the bactericidal activity of quaternary ammonium salts. J. Am. Chem. Soc..

[39] González-Pérez A., Czapkiewicz J., del Castillo J.L., Rodríguez J.R. (2001). Micellar properties of long-chain alkyldimethylbenzylammonium chlorides in aqueous solutions. Colloids Surf. Physicochem. Eng. Asp..

[40] Ledbetter J.W., Bowen J.R. (1969). Spectrophotometric determination of the critical micelle concentration of some alkyldimethylbenzylammonium chlorides using fluorescein. Anal. Chem..

[41] Klevens H.B. (1953). Structure and aggregation in dilate solution of surface active agents. J. Am. Oil Chem. Soc..

[42] Hage S.E., Lajoie B., Stigliani J.-L., Furiga-Chusseau A., Roques C., Baziard G. (2014). Synthesis, antimicrobial activity and physico-chemical properties of some *n*-alkyldimethylbenzylammonium halides. J. Appl. Biomed..

[43] Bisacchi G.S., Sutton J.C., Slusarchyk W.A., Treuner U., Zhao G. (2002). Beta Lactam Compounds and Their Use as Inhibitors of Tryptase. U.S. Patent.

[44] Brycki B., Kowalczyk I., Kozirog A. (2011). Synthesis, molecular structure, spectral properties and antifungal activity of polymethylene-α,ω-bis(*N*,*N*-dimethyl-*N*-dodecyloammonium bromides). Molecules.

[45] Koziróg A., Brycki B. (2015). Monomeric and gemini surfactants as antimicrobial agents—Influence on environmental and reference strains. Acta Biochim. Pol..

