# Supplementary material for: Synthesis, Structure and Antimicrobial Properties of Novel Benzalkonium Chloride Analogues with Pyridine Rings
Source: Molecules. 2017 Jan 13;22(1):130. doi: 10.3390/molecules22010130 (PMC6155866; doi:10.3390/molecules22010130)

Supplementary Materials: Synthesis, Structure and Antimicrobial Properties of Novel Benzalkonium Chloride Analogues with Pyridine Ring

Bogumił Brycki, Izabela Małecka, Anna Koziróg and Anna Otlewska

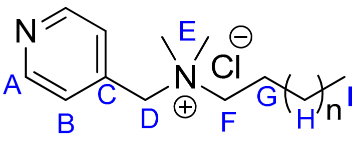

P13-P18

P13 n=5
P14 n=7
P15 n=9
P16 n=11
P17 n=13
P18 n=15

Table S1. Assignments of characteristic ^{1}H-NMR and ^{13}C-NMR signals of **P13–P18**.

	P13		P14		P15		P16		P17		P18	
	^{1}H	^{13}C	^{1}H	^{13}C	^{1}H	^{13}C	^{1}H	^{13}C	^{1}H	^{13}C	^{1}H	^{13}C
**A**	8.71	150.65	8.71	150.67	8.70	150.66	8.71	150.68	8.71	150.70	8.71	150.68
**B**	7.73	127.59	7.72	127.59	7.72	127.58	7.73	127.58	7.73	127.59	7.73	127.58
**C**	-	136.17	-	136.14	-	136.14	-	136.12	-	136.15	-	136.12
**D**	5.24	65.58	5.26	65.57	5.25	65.57	5.30	65.49	5.30	65.53	5.30	65.49
**E**	3.34	49.86	3.34	49.86	3.34	49.86	3.35	49.79	3.35	49.82	3.35	49.79
**F**	3.53	64.06	3.53	64.04	3.52	64.02	3.54	64.02	3.54	64.07	3.54	64.02
**G**	1.80	31.47	1.80	31.71	1.80	31.77	1.80	31.80	1.80	31.82	1.80	31.80
**I**	0.87	13.92	0.88	14.00	0.88	14.00	0.88	14.02	0.88	14.03	0.87	14.03

Table S2. ESI-MS characteristic *m*/*z* peaks of **P13–P18**.

Compound	ESI(+)MS		ESI(−)MS
	$[M]^{+}$	$[2M + Cl]^{+}$	$[M + 2Cl]^{-}$
**P13**	249	533	319
**P14**	277	589	347
**P15**	305	645	327
**P16**	333	701	403
**P17**	361	757	431
**P18**	389	813	459

Table S3. The FT-IR bands and assignments of **P13–P18**.

P13	P14	P15	P16	P17	P18	Proposed Assignments
3436	3439	3454	3451	3409	3401	ν O–H
3242	3245	3244	3244	-	3265	ν O–H
3057	3063	3062	3062	3053	3054	ν $C–H_{aromatic}$
-	3034	3035	3034	3035	-	
3027	3023	3026	3026	3022	3023	
3008	3005	-	-	3007	3005	
2977	2985	2983	2983	2979	2979	ν $C–H_{aliphatic}$
2957	2952	2950	2949	2952	2953	
2929	2921	2920	2915	2915	2915	
2858	2850	2850	2850	2851	2851	
1623	1619	1619	1619	-	1617	ν C=N, ν $C=C_{aromatic}$
1597	1601	1602	1602	1600	1600	ν C=N, ν $C=C_{aromatic}$
1562	1564	1564	1564	1565	1564	ν C=N, ν $C=C_{aromatic}$
1473	1470	1470	1470	1471	1472	δ $C–H_{aliphatic}$
1415	1415	1419	1419	1417	1415	δ C=N, $C=C_{aromatic}$
1351	1355	1355	1355	1354	1353	δ $C–H_{aromatic}$
1222	1227	1227	1227	1228	1228	δ $C–H_{aromatic}$
1069	1074	1074	1074	1073	1072	δ $C–H_{aromatic}$
1006	996	998	996	996	996	γ C=N, $C=C_{aromatic}$
728	728	729	728	727	728	γ CH_2

Copies of Spectra of P13–P18.

P13 ^{1}H NMR, 300 MHz, CDCl 3

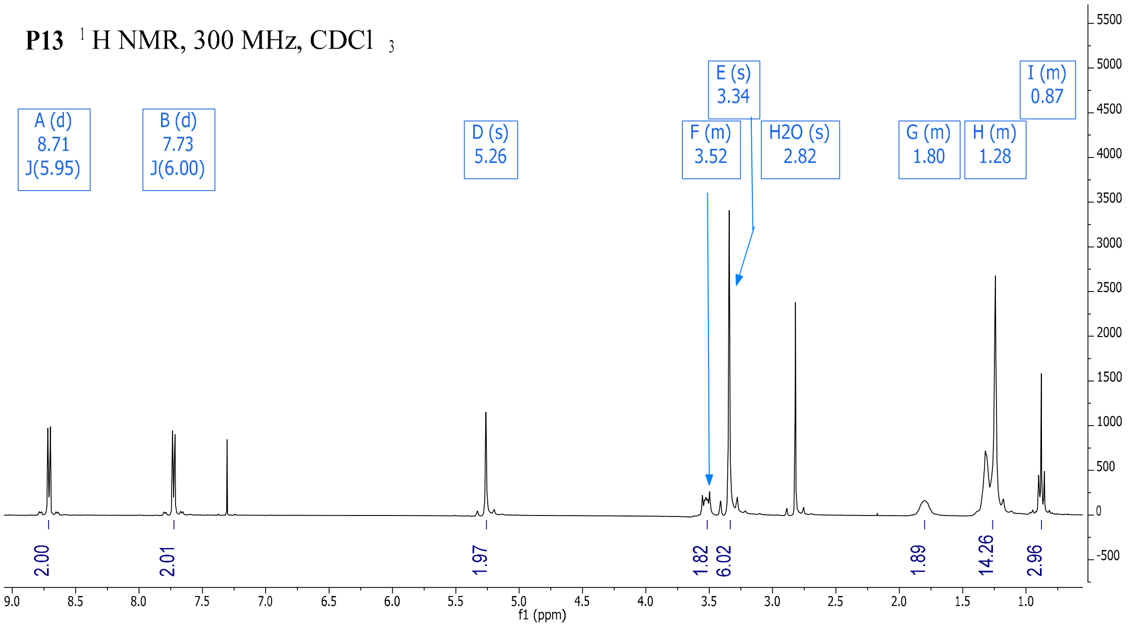

P13 ^{13}C NMR, 75 MHz, CDCl 3

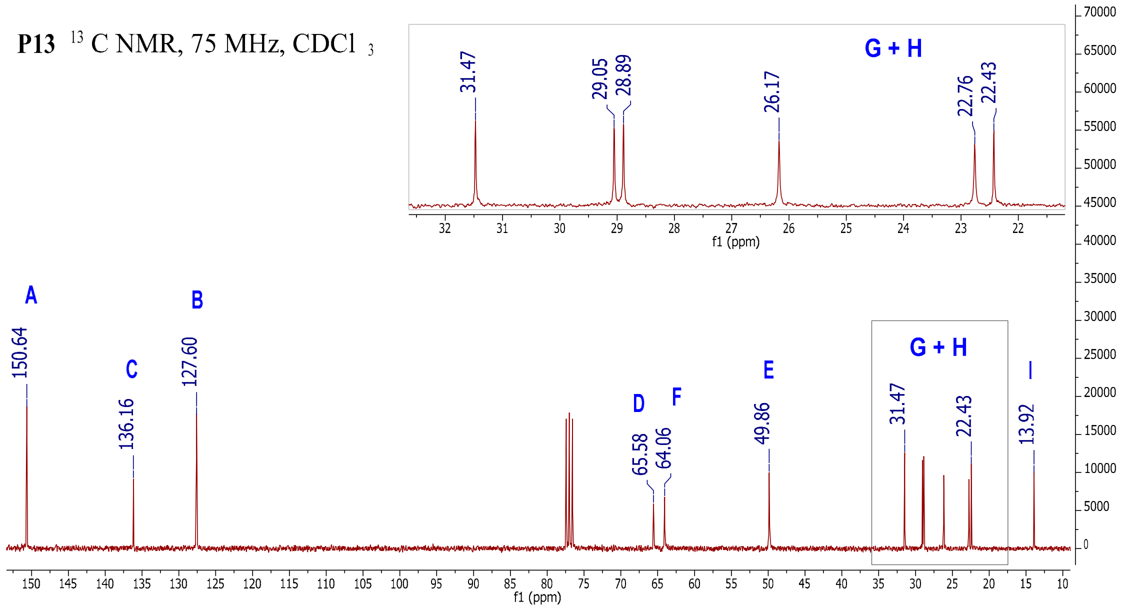

P13, FT-IR, KBr

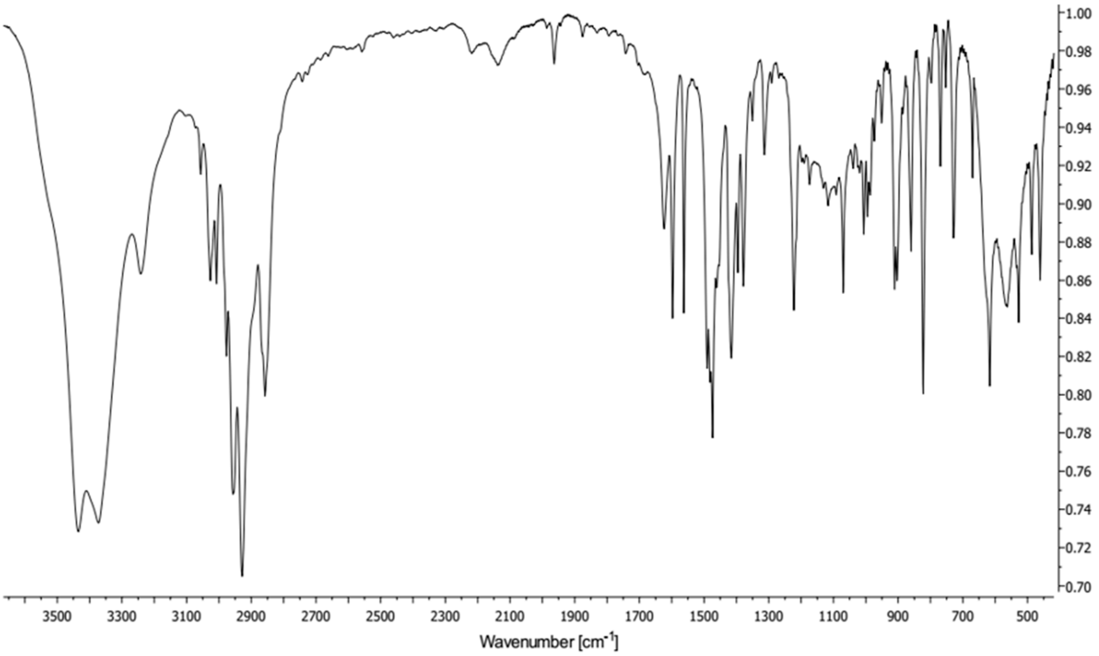

P13, ESI-MS, CH_3OH

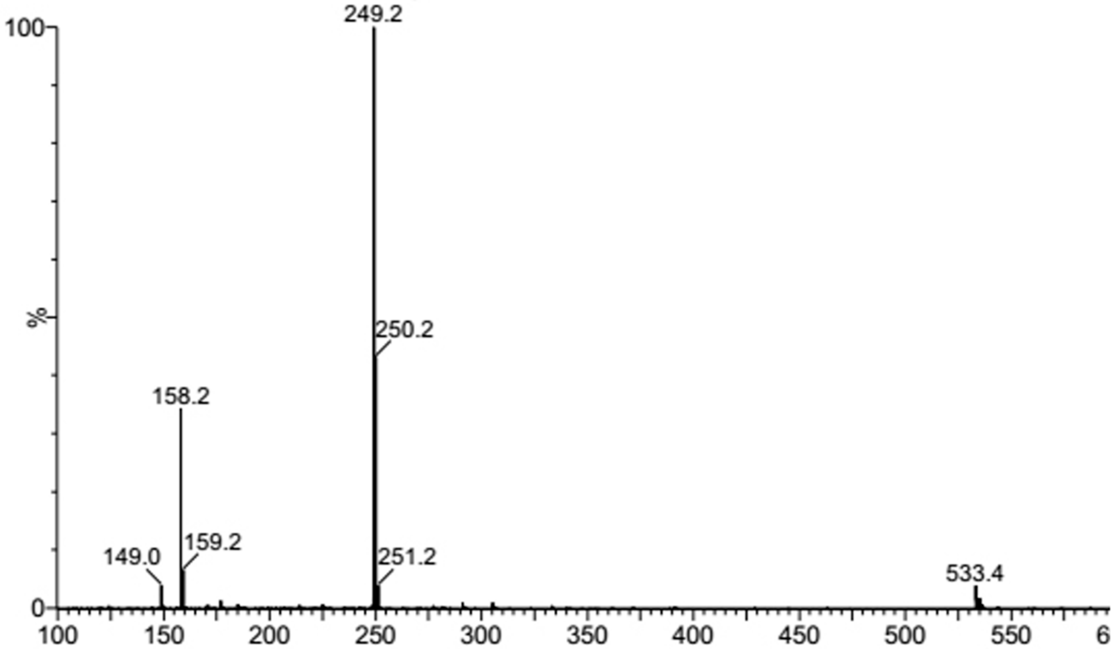

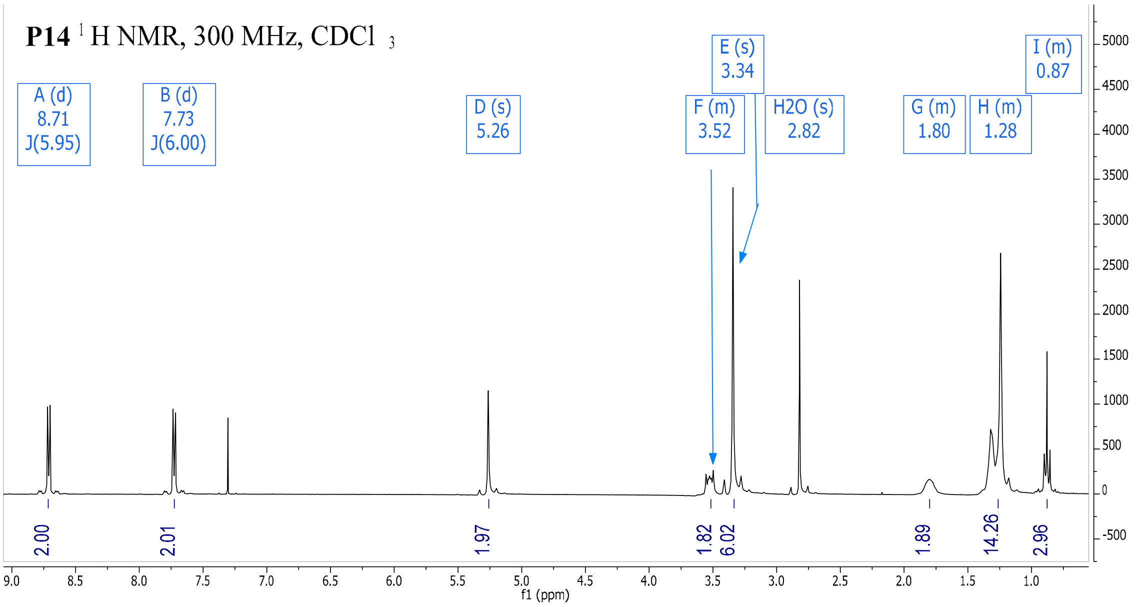
P14 [1] H NMR, 300 MHz, CDCl 3
A (d) 8.71 J(5.95)
B (d) 7.73 J(6.00)
D (s) 5.26
F (m) 3.52
E (s) 3.34
H2O (s) 2.82
G (m) 1.80
H (m) 1.28
I (m) 0.87
2.00
2.01
1.97
1.82
6.02
1.89
14.26
2.96
f1 (ppm)

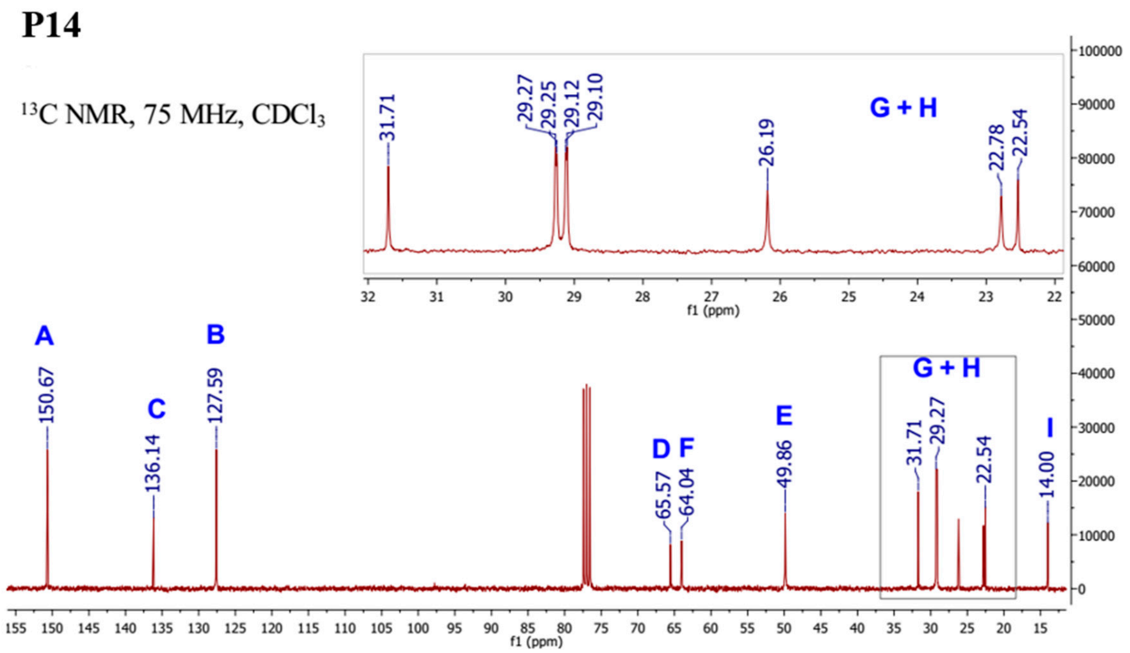
P14
13C NMR, 75 MHz, CDCl3
31.71
29.27
29.25
29.12
29.10
26.19
G + H
22.78
22.54
A
150.67
C
136.14
B
127.59
D F
65.57
64.04
E
49.86
G + H
31.71
29.27
22.54
I
14.00
f1 (ppm)

P14, ATR IR

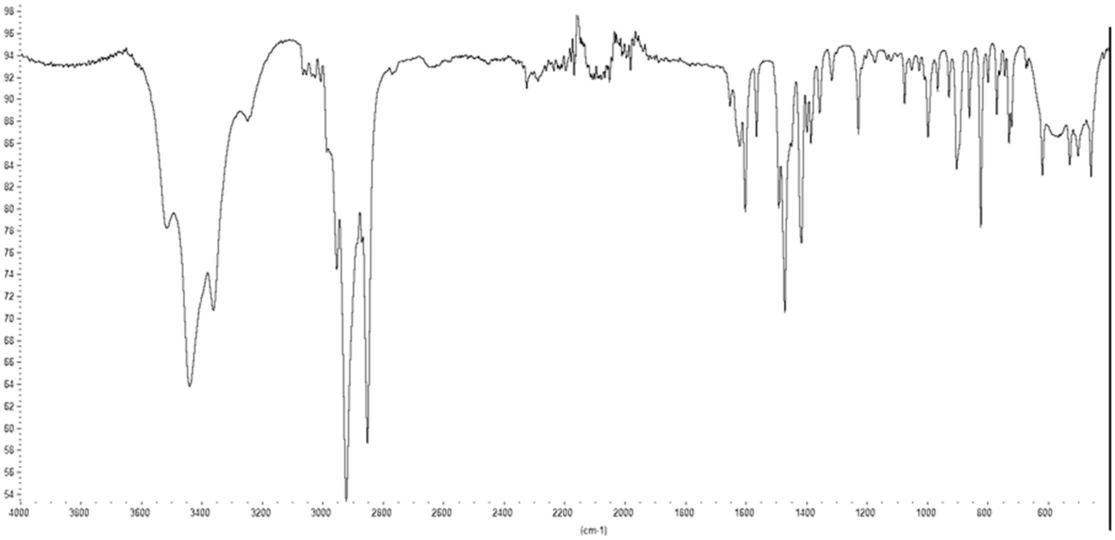

P14, ESI-MS, CH_3OH

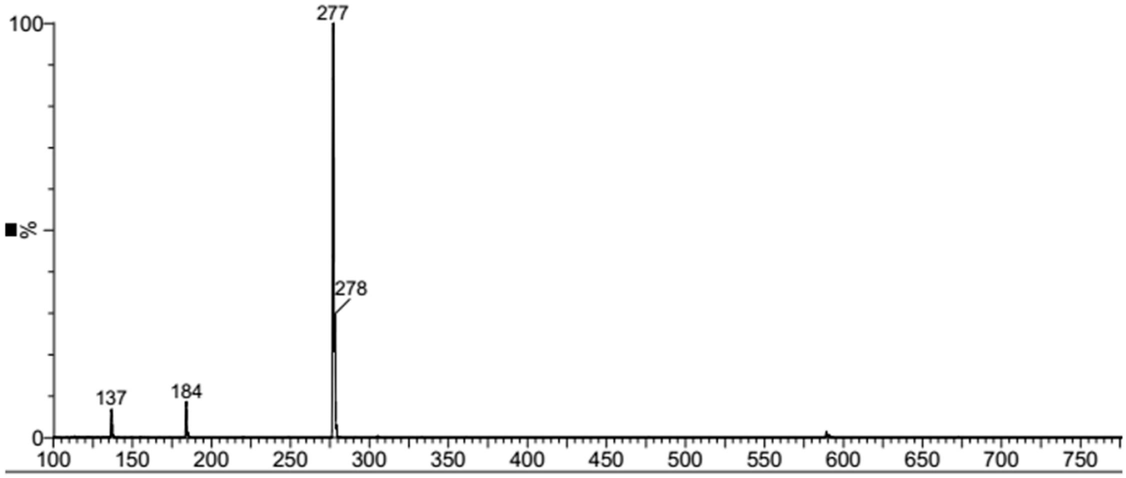

P15

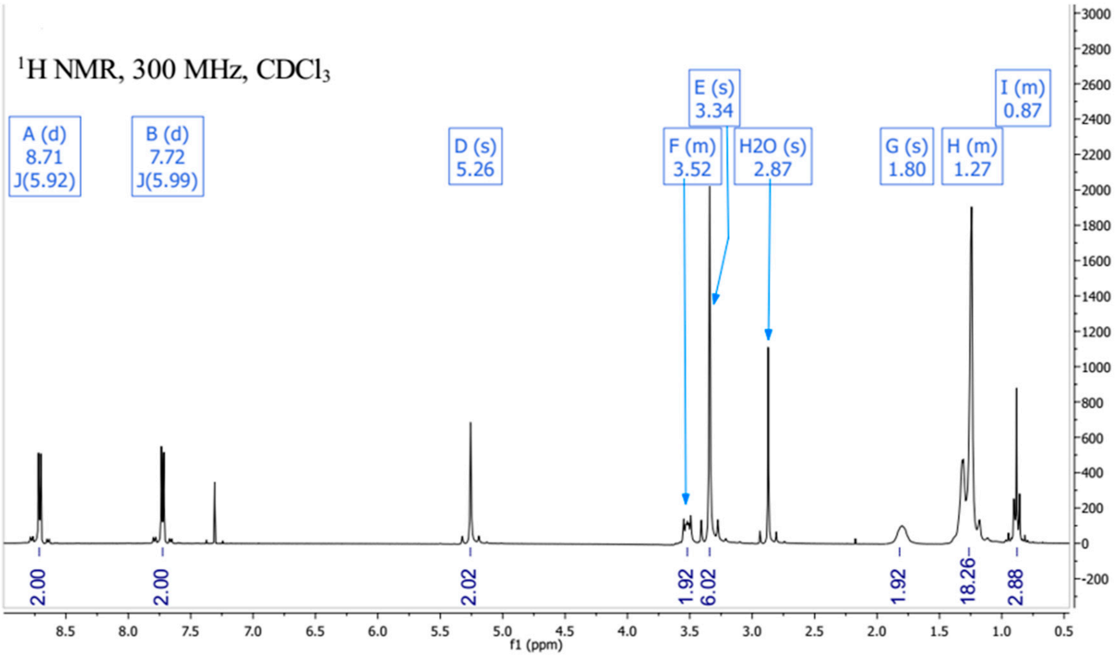

^{1}H NMR, 300 MHz, $CDCl_3$
A (d) 8.71 J(5.92)
B (d) 7.72 J(5.99)
D (s) 5.26
E (s) 3.34
F (m) 3.52
H2O (s) 2.87
G (s) 1.80
H (m) 1.27
I (m) 0.87
2.00
2.00
2.02
1.92
6.02
1.92
18.26
2.88
f1 (ppm)

P15

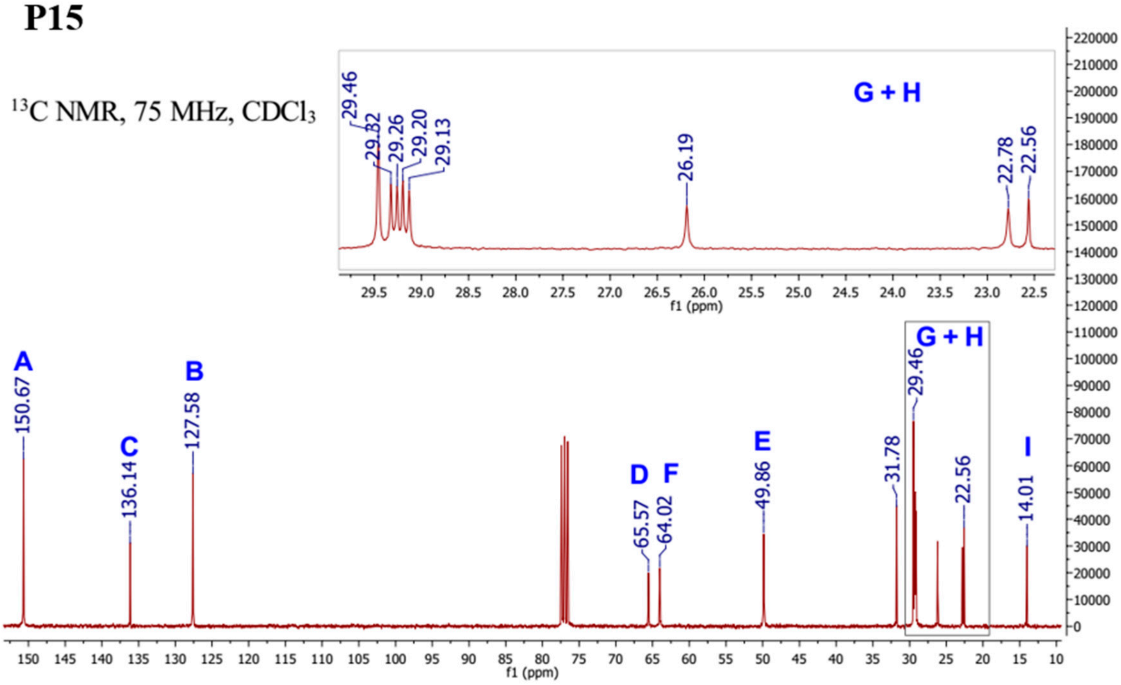

^{13}C NMR, 75 MHz, $CDCl_3$
G + H
29.46
29.32
29.26
29.20
29.13
26.19
22.78
22.56
f1 (ppm)
A 150.67
C 136.14
B 127.58
D 65.57
F 64.02
E 49.86
31.78
G + H 29.46
22.56
I 14.01
f1 (ppm)

P15, FT-IR, KBr

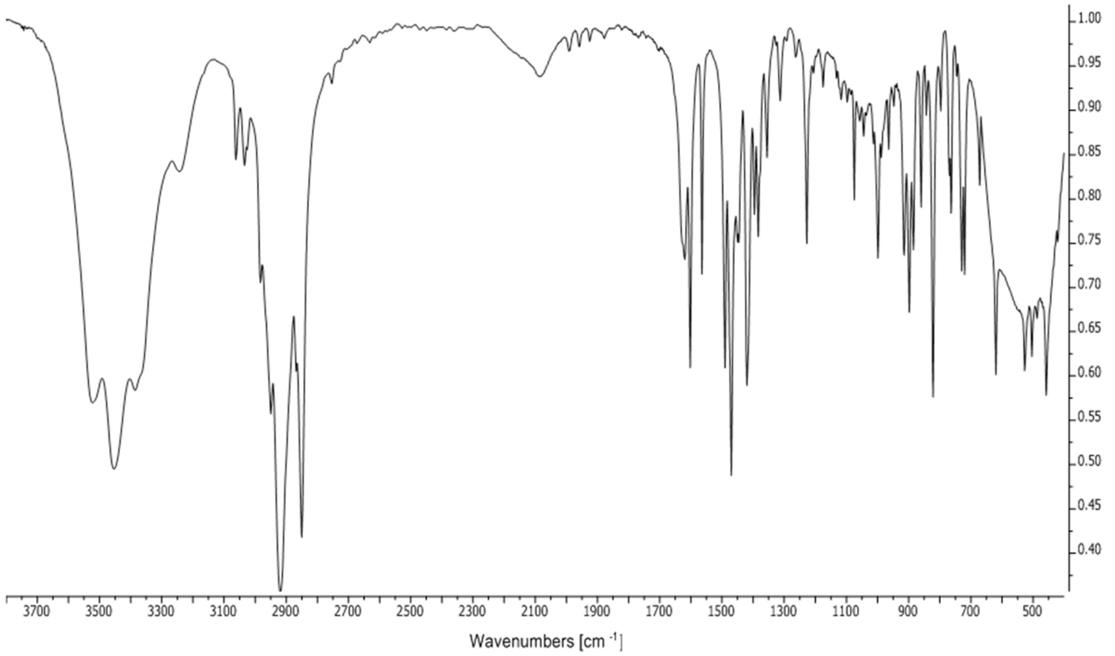

P15, ESI-MS, CH_3OH

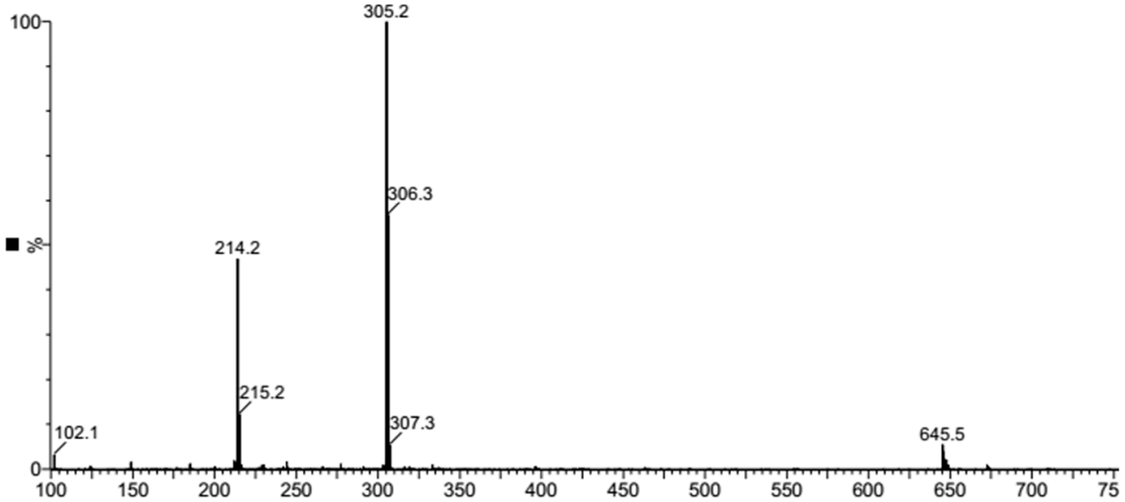

P16

^1H NMR, 403 MHz, $CDCl_3$

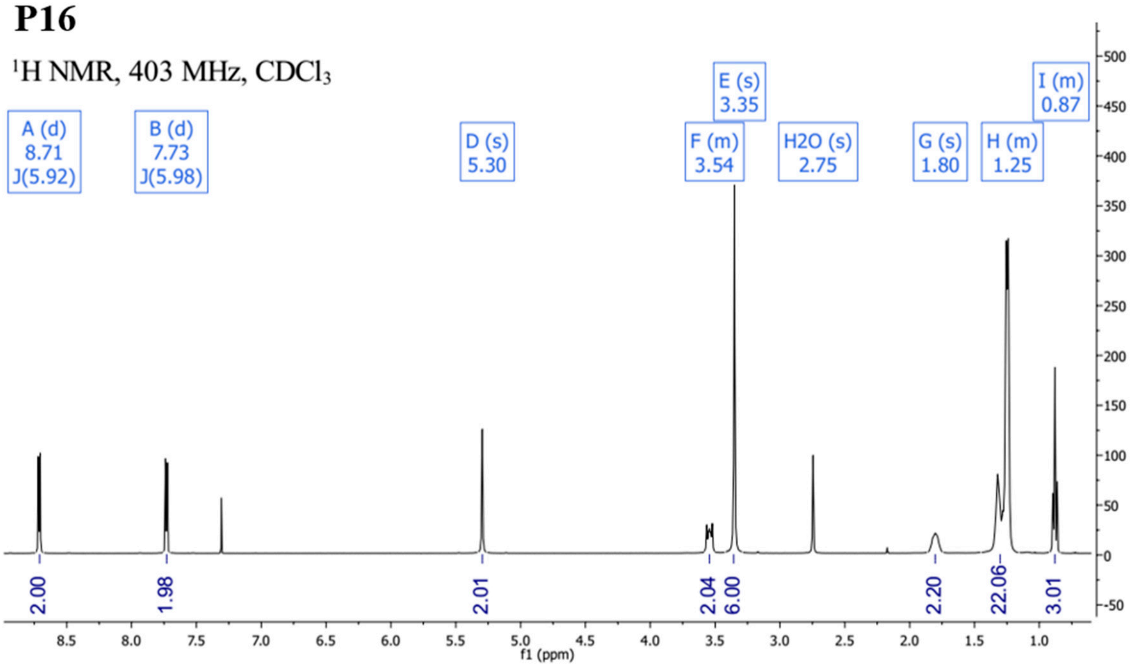

P16

^{13}C NMR, 101 MHz, $CDCl_3$

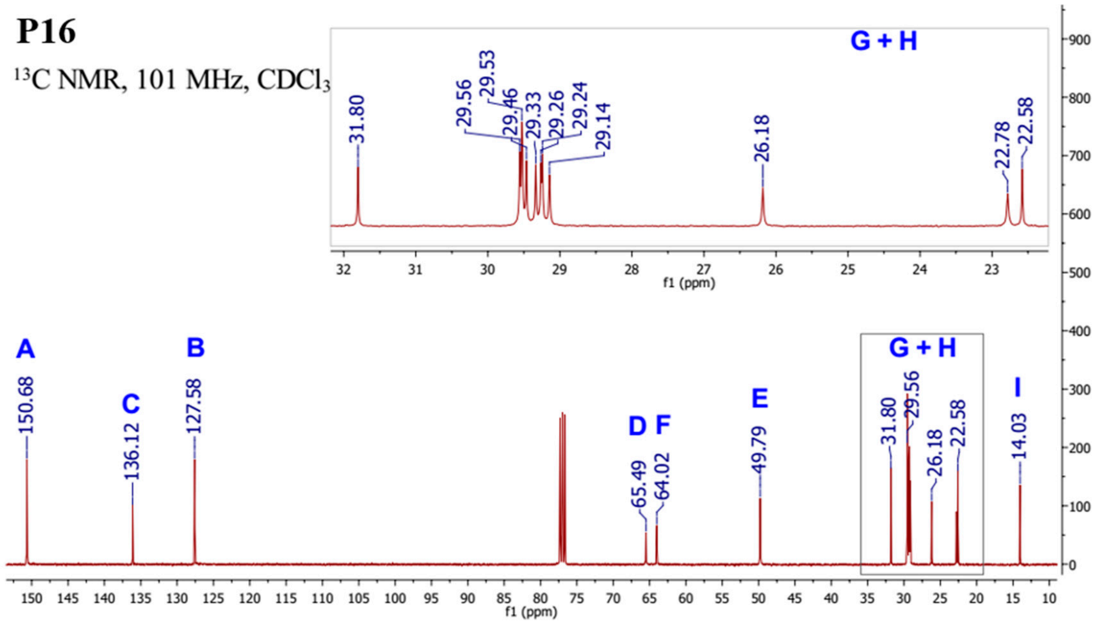

P16, FT-IR, KBr

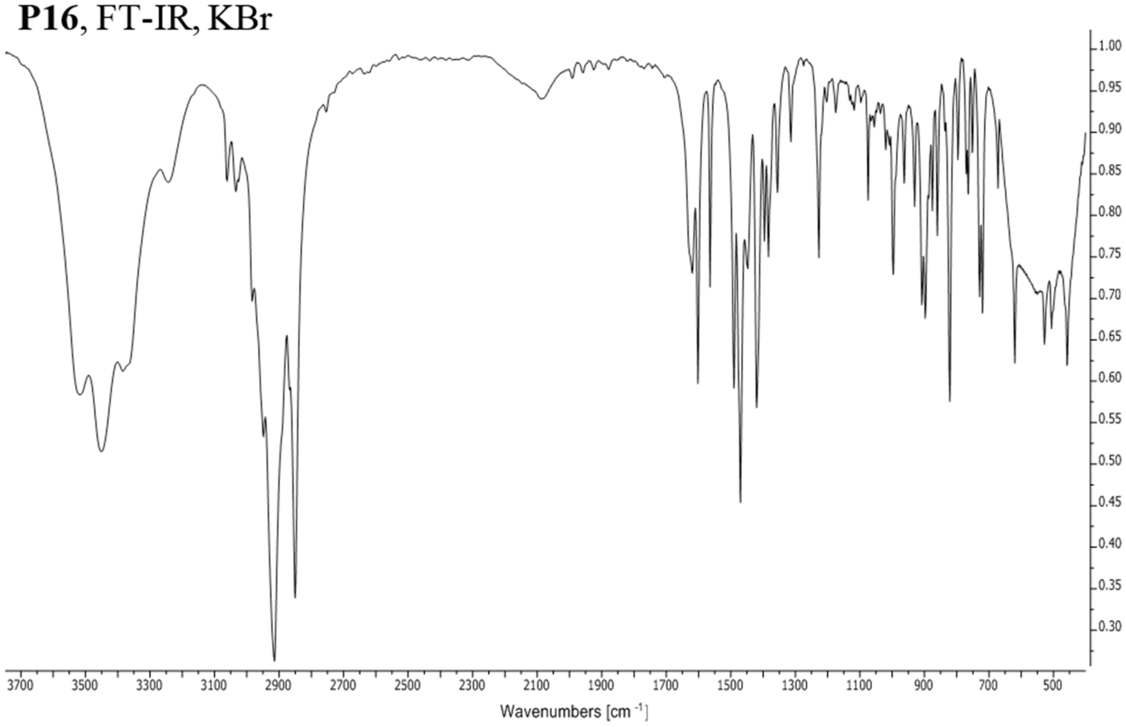

P16, ESI-MS, CH_3OH

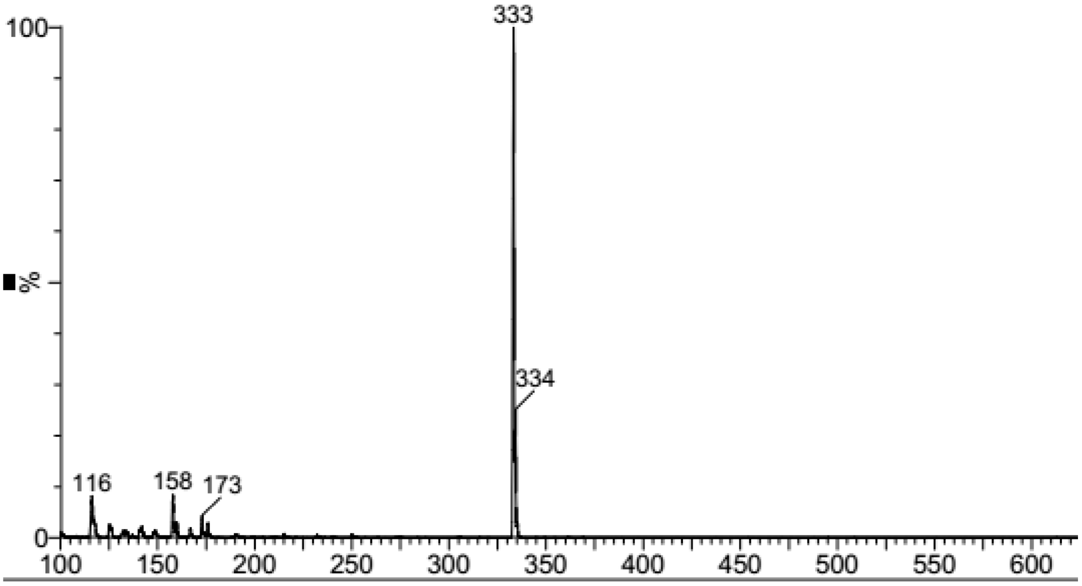

P17

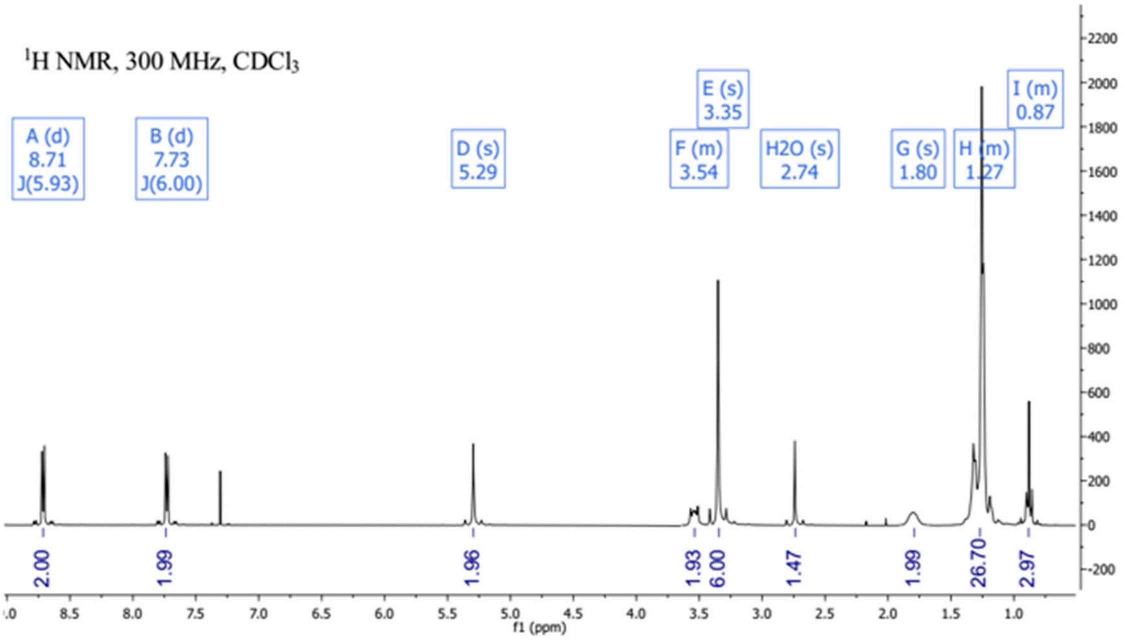

^{1}H NMR, 300 MHz, CDCl$_3$
A (d) 8.71 J(5.93)
B (d) 7.73 J(6.00)
D (s) 5.29
E (s) 3.35
F (m) 3.54
H2O (s) 2.74
G (s) 1.80
H (m) 1.27
I (m) 0.87
2.00
1.99
1.96
1.93
6.00
1.47
1.99
26.70
2.97
f1 (ppm)

P17

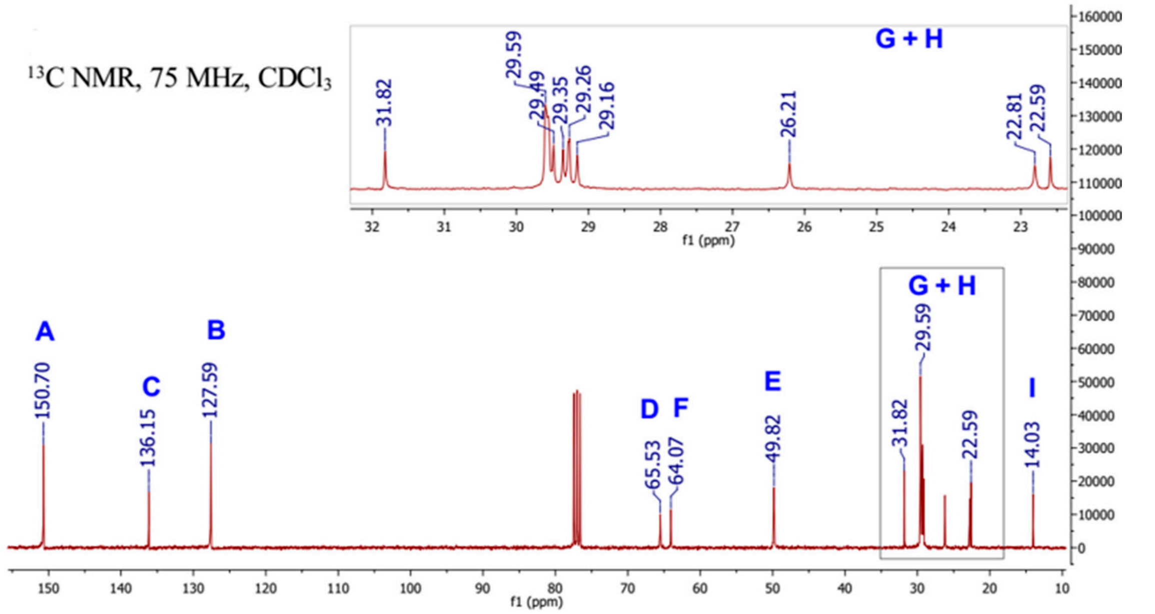

^{13}C NMR, 75 MHz, CDCl$_3$
G + H
31.82
29.59
29.49
29.35
29.26
29.16
26.21
22.81
22.59
A
150.70
C
136.15
B
127.59
D F
65.53
64.07
E
49.82
31.82
29.59
22.59
I
14.03
f1 (ppm)

P17, ATR IR

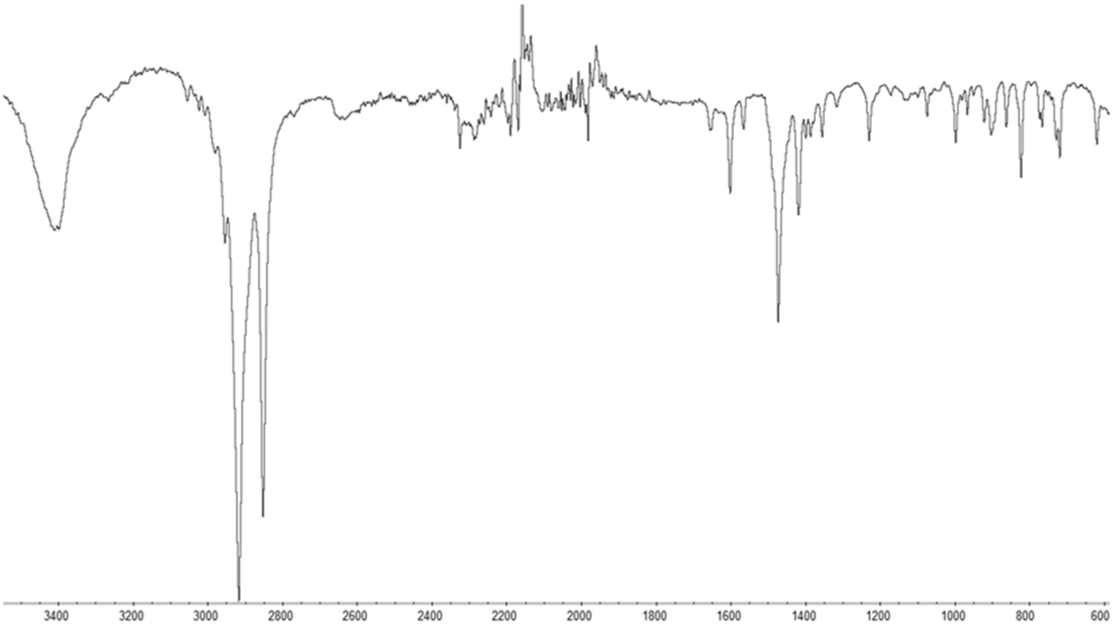

P17, ESI-MS

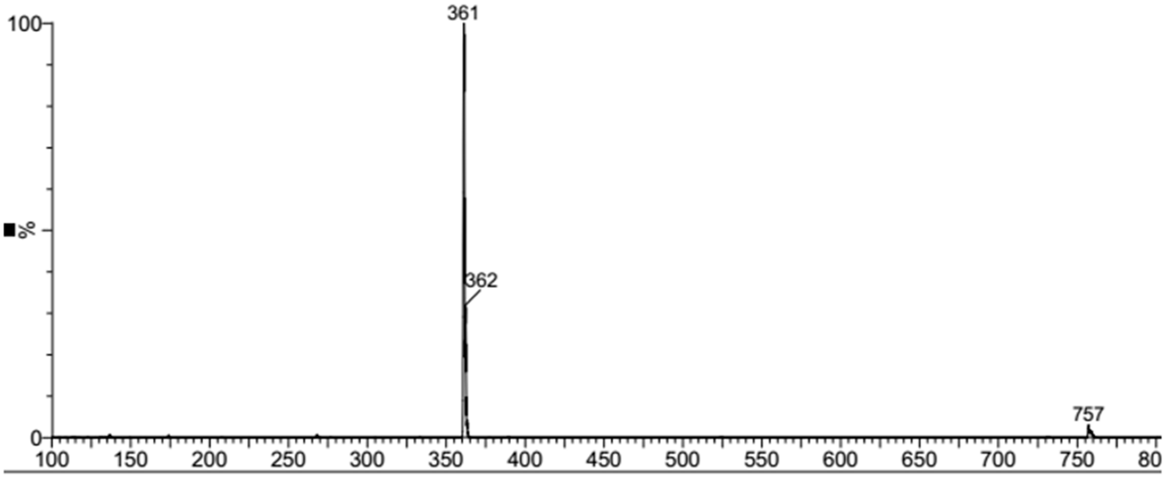

P18

^{1}H NMR, 403 MHz, $CDCl_3$

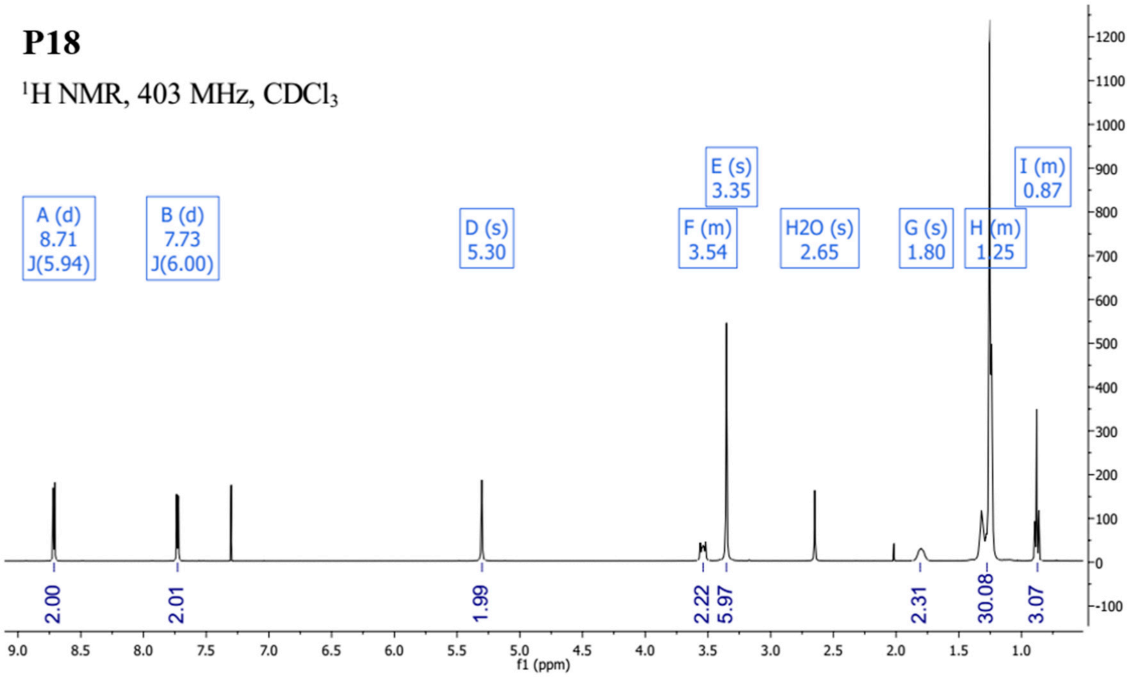

P18

^{13}C NMR, 101 MHz, $CDCl_3$

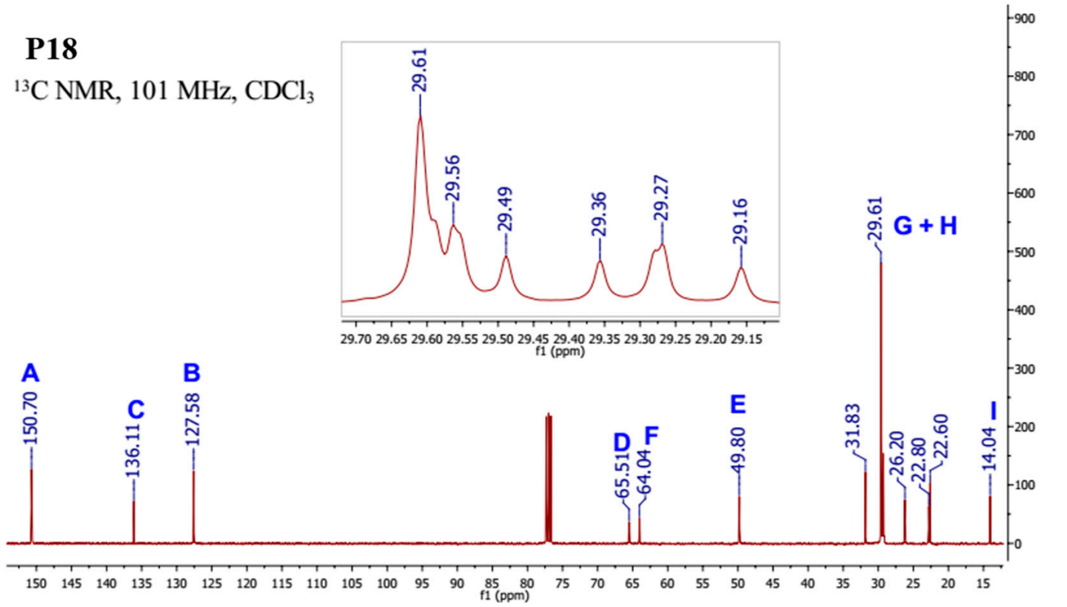

P18, FT-IR, KBr

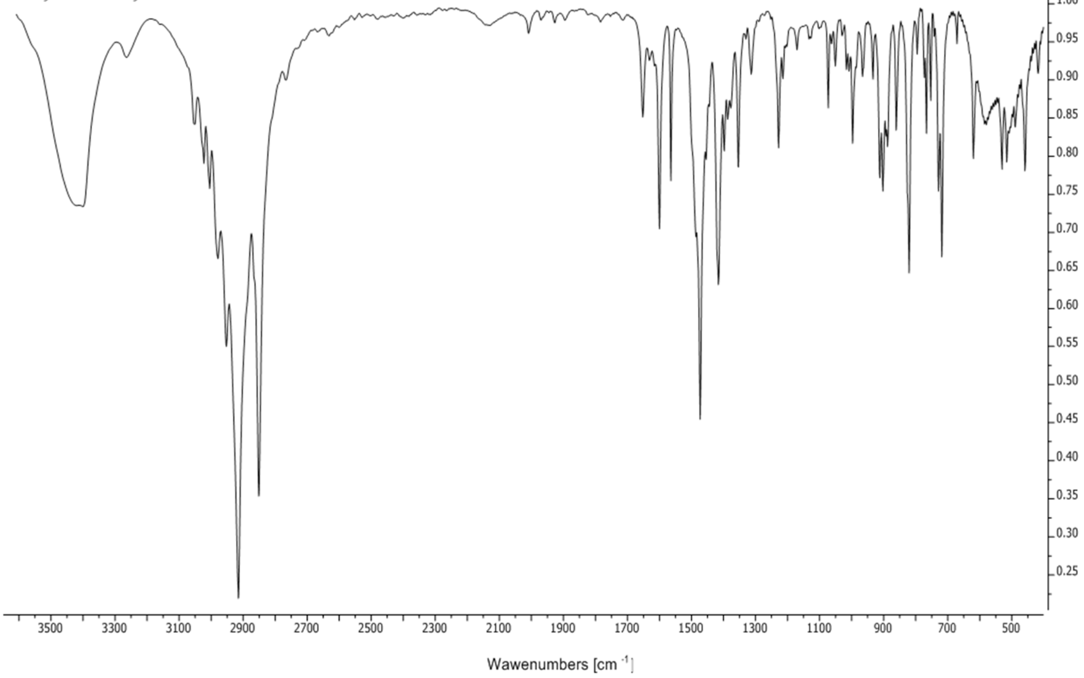

P18, ESI-MS, CH_3OH

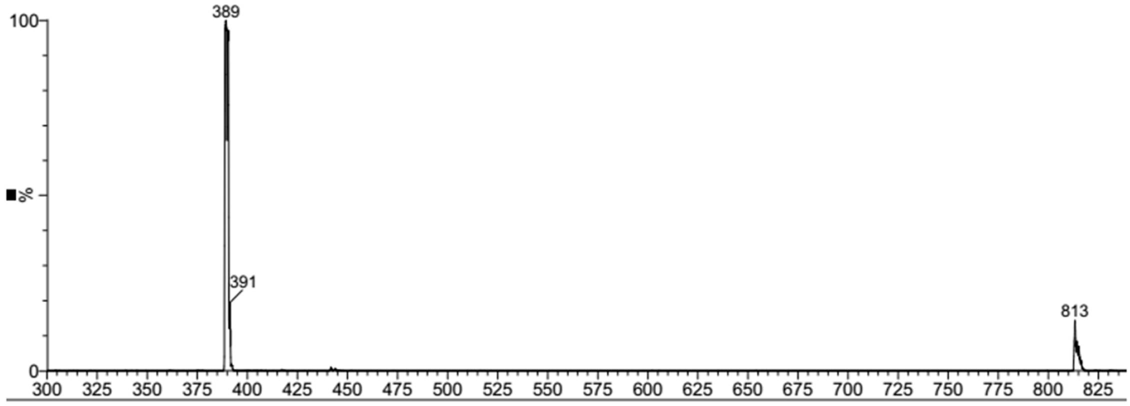

Conductometric Study of P14–P18.

**Compound P16**	Value	Standard Error
A1	84216,43962	2067,35963
A2	37885,73385	1360,02386
**x0**	**0,0049**	**5,71118E-5**
dx	2,86509E-4	5,2276E-5

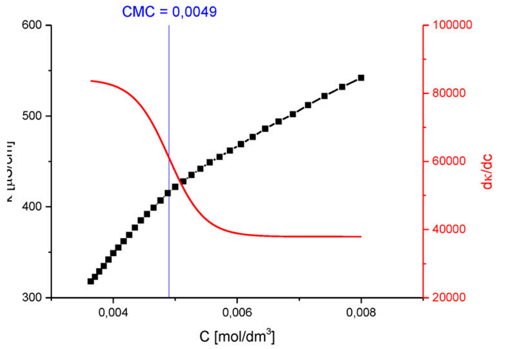

**Compound P16**	Value	Standard Error
A1	88576,57033	1470,30562
A2	37160,81642	792,04656
**x0**	**0,00483**	**3,4686E-5**
dx	3,26903E-4	3,1691E-5

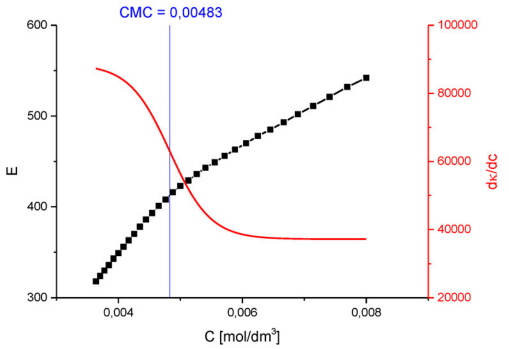

**Compound P15**	Value	Standard Error
A1	78957,20152	996,01981
A2	39902,77242	1066,76532
**x0**	**0,02027**	**1,43103E-4**
dx	0,00115	1,36593E-4

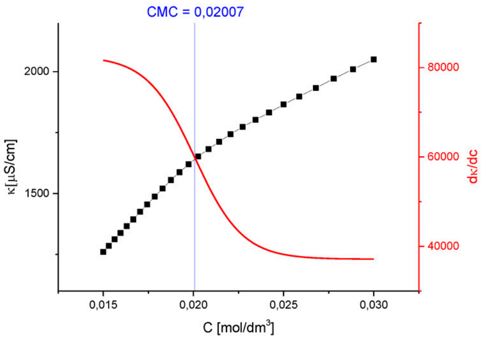

**Compound P15**	Value	Standard Error
A1	82614,71414	777,75371
A2	37119,62552	525,87247
**x0**	**0,02007**	**8,83459E-5**
dx	0,00133	8,34045E-5

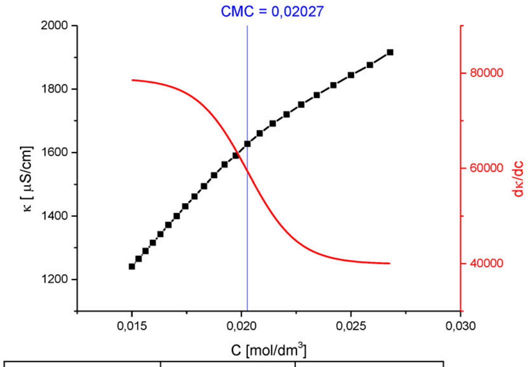

**Compound P17**	Value	Standard Error
A1	85144,24464	607,75447
A2	46071,13243	571,94527
**x0**	**0,00114**	**1,11484E-5**
dx	1,16097E-4	9,89944E-6

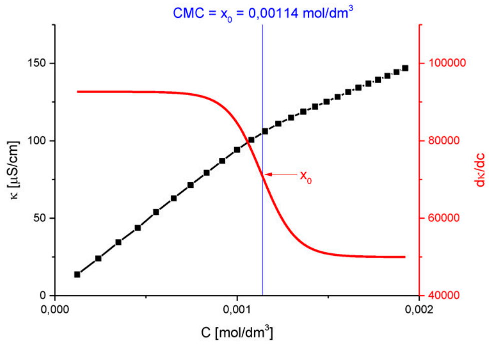

**Compound P17**	Value	Standard Error
A1	92623,81494	697,83205
A2	49978,01389	639,27552
**x0**	**0,00114**	**1,06549E-5**
dx	9,30534E-5	9,31556E-6

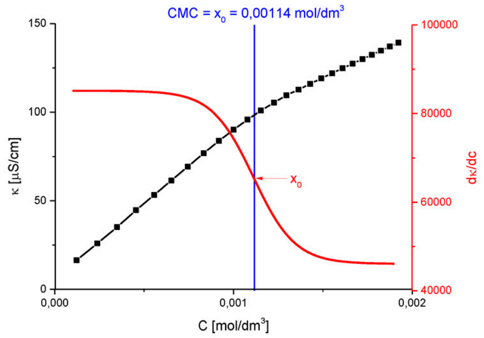

**Compound P18**	Value	Standard Error
A1	90681,71378	588,94026
A2	62250,10852	847,88225
**x0**	**3,11845E-4**	**5,38922E-6**
dx	5,25826E-5	5,15337E-6

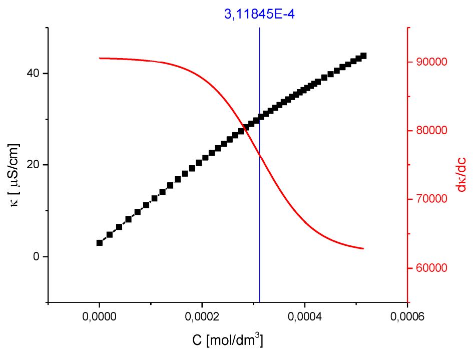

**Compound P18**	Value	Standard Error
A1	92015,63571	1112,604
A2	60235,52404	2033,93098
**x0**	**3,10843E-4**	**1,1927E-5**
dx	5,997E-5	1,16784E-5

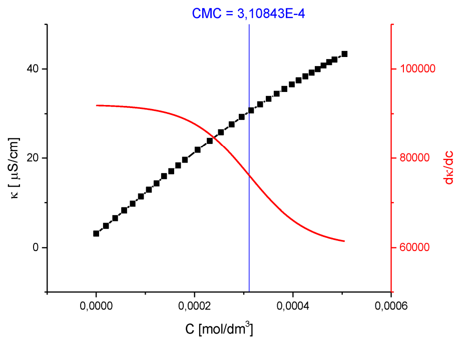

**Compound P14**	Value	Standard Error
A1	70,21248	4,34164
A2	36,63556	20,55195
**x0**	**0,08791**	**0,01989**
dx	0,01879	0,01289

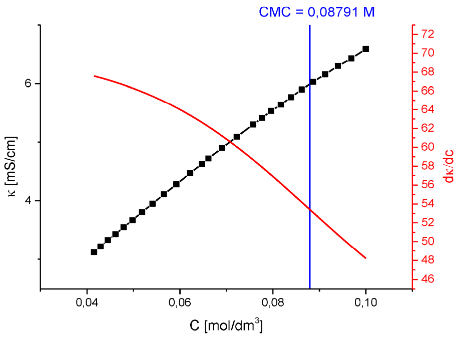

**Compound P14**	Value	Standard Error
A1	62,87987	3,53237
A2	45,03613	8,77492
**x0**	**0,08633**	**0,00675**
dx	0,00679	0,00667

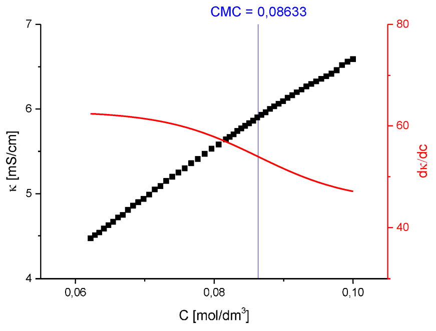

Supplement: Supplementary file 1 [file molecules-22-00130-s001.pdf]
